# Diagnostic accuracy of prenatal imaging for the diagnosis of congenital Zika syndrome: Systematic review and meta-analysis

**DOI:** 10.3389/fmed.2022.962765

**Published:** 2022-09-29

**Authors:** Tania T. Herrera, Idalina Cubilla-Batista, Amador Goodridge, Tiago V. Pereira

**Affiliations:** ^1^Centro de Investigación Médica Pacifica Salud-INDICASAT AIP, Pacifica Salud, Hospital Punta Pacicifica, Panama City, Panama; ^2^Centro de Biología Celular y Molecular de Enfermedades-Instituto de Investigaciones Científicas y Servicios de Alta Tecnología (INDICASAT-AIP), City of Knowledge, Panama City, Panama; ^3^Hospital Rafael Estévez, Caja de Seguro Social, Aguadulce, Panama; ^4^Department of Health Sciences, College of Medicine, University of Leicester, Leicester, United Kingdom

**Keywords:** congenital Zika syndrome (CZS), Zika virus infection, pregnancy, neurosonology, ventriculomegaly, ZIKV, microcephaly

## Abstract

**Objective:**

The aim of this study was to assess the accuracy of prenatal imaging for the diagnosis of congenital Zika syndrome.

**Data sources:**

Medline (via Pubmed), PubMed, Scopus, Web of Science, and Google Scholar from inception to March 2022. Two researchers independently screened study titles and abstracts for eligibility.

**Study eligibility criteria:**

Observational studies with Zika virus-infected pregnant women were included. The index tests included ultrasound and/or magnetic resonance imaging. The reference standard included (1) Zika infection-related perinatal death, stillbirth, and neonatal death within the first 48 h of birth, (2) neonatal intensive care unit admission, and (3) clinically defined adverse perinatal outcomes.

**Synthesis methods:**

We extracted 2 × 2 contingency tables. Pooled sensitivity and specificity were estimated using the random-effects bivariate model and assessed the summary receiver operating characteristic (ROC) curve. Risk of bias was assessed using QUADAS 2 tool. The certainty of the evidence was evaluated with grading of recommendations.

**Results:**

We screened 1,459 references and included 18 studies (2359 pregnant women, 347 fetuses with confirmed Zika virus infection). Twelve studies (67%) were prospective cohorts/case series, and six (37%) were retrospective cohort/case series investigations. Fourteen studies (78%) were performed in endemic regions. Ten studies (56%) used prenatal ultrasound only, six (33%) employed ultrasound and fetal MRI, and two studies (11%) used prenatal ultrasound and postnatal fetal MRI. A total of six studies (ultrasound only) encompassing 780 pregnant women (122 fetuses with confirmed Zika virus infection) reported relevant data for meta-analysis (gestation age at which ultrasound imagining was captured ranged from 16 to 34 weeks). There was large heterogeneity across studies regarding sensitivity (range: 12 to 100%) and specificity (range: 50 to 100%). Under a random-effects model, the summary sensitivity of ultrasound was 82% (95% CI, 19 to 99%), and the summary specificity was 97% (71 to 100%). The area under the ROC curve was 97% (95% CI, 72 to 100%), and the summary diagnostic odds ratio was 140 (95% CI, 3 to 7564, *P* < 0.001). The overall certainty of the evidence was “very low”.

**Conclusion:**

Ultrasound may be useful in improving the diagnostic accuracy of Zika virus infection in pregnancy. However, the evidence is still substantially uncertain due to the methodological limitations of the available studies. Larger, properly conducted diagnostic accuracy studies of prenatal imaging for the diagnosis of congenital Zika syndrome are warranted.

**Systematic review registration:**

Identifier [CRD42020162914].

## Introduction

Zika virus (ZIKV) infection is an emerging public health problem in several regions worldwide. Zika virus (ZIKV) was first isolated in 1947 from a sentinel monkey in the Zika forest in Uganda, Africa (1). Following an outbreak in the Americas in 2015, the virus spread across 59 countries, and transmission still persists in many countries with more than 500,00 suspected cases reported (2–6).

The rate of maternal–fetal transmission ranges from 7 to 18% in confirmed or probable cases (7). A recent study in Brazil reported a rate as high as 35% (8). ZIKV perinatal infection causes neurological malformations that characterize congenital Zika syndrome (CZS) (3–5). The ZIKV strains, belonging to the Asian lineage, replicate in the placenta and fetal brain (9–11). In the brain, the virus infects neuronal progenitor cells, resulting in the inhibition of cell proliferation and differentiation, as well as neuronal apoptosis (12). CZS includes few developmental and morphological hallmarks, including but not limited to microcephaly, agenesia/hypoplasia of the corpus callosum, parenchymal calcifications, brain stem hypoplasia, and ocular injury (4, 9, 11, 13). Neonatal findings range from cognitive, sensory, and motor disabilities to macular scarring and focal pigmentary retinal mottling; marked early hypertonia and extrapyramidal symptoms may also be present (3–7).

ZIKV infection diagnosis is marked by unique challenges that affect accuracy. According to the Center for Disease Control (CDC) guidelines, ZIKV RNA amplification by reverse transcription-polymerase chain reaction (RT-PCR) should be performed in serum, blood, or urine as early as possible and up to 12 weeks after symptom onset (CDC guidelines). Diagnosing ZIKV infection is complicated for several reasons: the mother is usually asymptomatic or shows non-specific symptoms (7). ZIKV has a narrow window of viremia and viruria, making RT-PCR detection difficult, and diagnostic tests can show cross-reactivity between ZIKV and dengue virus (14, 15). A recent systematic review indicated that ZIKV RNA could be cleared from amniotic fluid during pregnancy. Therefore, a negative ZIKV test result from amniocentesis did not provide adequate reassurance that the fetus was unaffected (16). The optimal timing for performing amniocentesis and the positive predictive value of amniocentesis for congenital ZIKV syndrome have not yet been established (16, 17).

The CDC, Society of Maternal-Fetal Medicine, the American College of Obstetricians and Gynecologists (ACOG), and International Society of Ultrasound in Obstetrics and Gynecology (ISUOG) all recommend ultrasound (US) and neurosonology as the preferred tool to evaluate the fetal brain and screen for other fetal anomalies (12–15, 17–20).

Fetal magnetic resonance imaging (MRI) has increased the positive predictive value of fetal brain abnormalities; however, it has a low negative predictive value and is associated with a greater number of false positives (21).

In a recent study investigating the rate of adverse outcomes in proven infected fetuses/newborns, 45% of the cases presented no signs of complications. Also, 20% had mild/moderate signs potentially correlated with congenital ZIKV (cZIKV) infection, 21% had severe complications, and 14% resulted in fetal loss (7).

*The objective of this systematic review was to identify the diagnostic accuracy of prenatal imaging studies for perinatal outcomes of fetuses exposed to maternal ZIKV infection.*

## Methods

This systematic review was conducted following the “Synthetizing Evidence from Diagnostic Accuracy Test (SEDATE) guidelines” (Supplementary Table 1) (22) and reported in agreement with the “Preferred Reporting Items for Systematic Reviews and Meta-Analyses (PRISMA-DTA statement guidelines)” (Supplementary Table 2) (23).

The study protocol was prospectively registered at the International Prospective Register PROSPERO (number: CRD42018097200) for quality control (24).

### Study selection process

We included observational cohort studies (retrospective and prospective) of pregnant women with ZIKV infection in any trimester of pregnancy whose fetuses developed CZS and underwent prenatal imaging studies (USG and/or MRI) and clinical follow-up of the neonate. We selected studies that determined ZIKV infection through real-time PCR when possible, immunoglobulin M (IgM) serological tests, and/or plaque neutralization reduction tests (PRNT). Exclusion criteria included case series with less than four cases, pediatric series on newborns and children from which maternal and pregnancy information could not be retrieved. We also excluded studies with insufficient outcome data.

## Index test

We considered ultrasound and fetal magnetic resonance as index tests, either alone or in combination.

### Reference standard test

The lack of a gold standard reference test is an acknowledged problem in studies of CZS. Reference tests for the diagnosis of CZS have important limitations (25).

For the present review, infants with a positive Zika RT-PCR from at least one fetal or neonatal sample (cerebrospinal fluid, amniotic fluid, placental tissue, or fetal tissue) were defined as confirmed CZS cases. Infants with Zika virus IgM detected in umbilical cord/neonatal blood or in cerebrospinal fluid were also considered confirmed cases. When performed, amniocentesis was used to rule outinfections such as toxoplasmosis, syphilis, rubella, cytomegalovirus and herpes simplex or genetic causes for congenital anomalies. We also considered as reference standard clinical examinations performed at birth by neonatologists, pediatric infectious disease specialists, and geneticists. A fetus or neonate with severe microcephaly, cortical hypoplasia with abnormal gyral patterns, intracranial calcifications located between cortex or subcortex, congenital contractures, or macular scarring was considered as confirmed CZS case. We also considered as confirmed CZS cases perinatal death, including stillbirth and neonatal death within the first 48 h after birth; admission to the neonatal intensive care unit (NICU); composite adverse perinatal outcomes as defined by each individual study; and abnormal clinical follow-up, which included seizures, eye abnormalities, hearing abnormalities, and abnormal neurological examinations that included postnatal imaging, such as transcranial ultrasound and computerized tomography scan.

In this study, the reference standard is composed of

1. Perinatal death, including stillbirth and neonatal death within the first 48 h after birth;
2. Admission to neonatal intensive care unit (NICU);
3. Composite adverse perinatal outcomes according to the definition of each individual study, which commonly includes any adverse outcomes occurring in the studied population; and/or
4. Abnormal clinical follow-up included seizures, eye abnormalities, hearing abnormalities, and abnormal neurological examinations.

### Data extraction

We piloted the screening strategy and the data extraction sheet. Two investigators (TH and AG) extracted data from eligible studies independently. We used a modified extraction template obtained from the Cochrane community web site (26) including the following study characteristics: authors, year of publication, settings, country, study design, period when the study was conducted, inclusion criteria, sample size, patient characteristics (inclusion and exclusion criteria, demographics), how the test was performed (gestational age at testing), and the type of reference standard test. We carefully cross-checked references for overlapping studies and solved all discrepancies between assessors via consensus, or consultation with a third investigator (ICB).

### Assessment of risk of bias

Assessments were conducted by a pair of reviewers (TH and AG) working independently. Discrepancies were resolved by consensus or discussion with a third investigator. We used the QUADAS 2 tool, which evaluates the risk of bias and applicability of the tests. QUADAs 2 has four domains: patient selection, index test, reference standard, and flow and timing (27). Each domain had signaling questions that were categorized as low, high, or unclear risk of bias (Supplementary Table S3). We used the risk of bias assessment to judge the certainty of the overall evidence using the Grading of Recommendations Assessments, Development and Evaluation (GRADE) system (28).

### Strategy for data synthesis and statistical analysis

A template for 2 × 2 tables describing the true positive (TP), false positive (FP), false negative (FN), and true negative (TN) results for each included study was constructed. We performed a meta-analysis assuming a random-effect model to obtain summary diagnostic accuracy estimates (and their 95% CIs). We used both bivariate and hierarchical summary receiver operating characteristic (HSROC) meta-analysis models (29–31). Summary HSROC curves with 95% confidence and 95% prediction regions were presented. The latter reflects the between-study heterogeneity, indicating the approximate predictive distribution of accuracy measures to be estimated in a future diagnostic accuracy study. The random-effects summary diagnostic odds ratio (DOR) was also calculated as a single metric that combines sensitivity and specificity (29, 31). A DOR > 1 captures how many times the chance of a positive US finding is higher among ZIKV-infected pregnant women than among pregnant women without Zika infection.

The statistical heterogeneity across sensitivity and specificity measures was assessed by Cochran’s *Q*-test and quantified with the I^2^ statistic. For Cochran’s *Q*-test, a *p*-value < 0.10 was considered evidence of variability across studies larger than expected by chance (31). For the I^2^ statistic, values > 50% were considered evidence of substantial statistical heterogeneity (30). Deek’s test was used to evaluate funnel plot asymmetry (31). We also plotted the log(DOR) against the inverse squared root of the effective sample size for visual inspection of funnel plot asymmetry. A *p*-value < 0.10 was deemed evidence of funnel plot asymmetry. All analyses were conducted in Stata (version 14, College Station, TX, United States).

## Results

### Study selection and study characteristics

**FIGURE 1:**
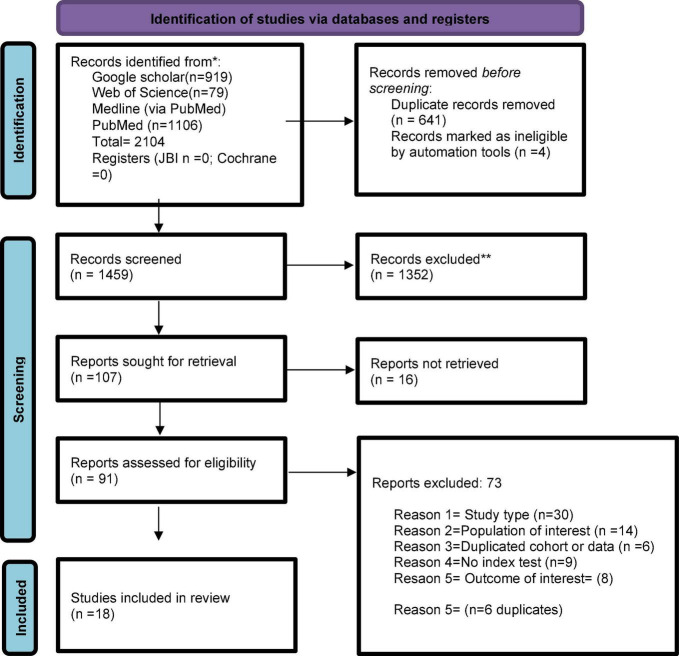
Flowchart summarizing inclusion in systematic review of studies. *Consider, if feasible to do so, reporting the number of records identified from each database or register searched (rather than the total number across all databases/registers). **If automation tools were used, indicate how many records were excluded by a human and how many were excluded by automation tools. *From:* Page MJ, McKenzie JE, Bossuyt PM, Boutron I, Hoffmann TC, Mulrow CD, et al. The PRISMA 2020 statement: an updated guideline for reporting systematic reviews. BMJ 2021;372:n71. doi: 10.1136/bmj.n71. For more information, visit: http://www.prisma-statement.org/.

Figure 1 shows the study selection process. We identified 1459 citations. After removing duplicates, we assessed 91 full-length studies, of which 18 were included in our systematic review. Supplementary Table S4 summarizes the list of excluded studies with reasons for their exclusion.

A total of 347 fetuses *with confirmed Zika* Virus infection were investigated across the 18 included studies. The pregnancy termination rate after fetal diagnosis of CZS was low, with only 36 (1.5%) women choosing to have an abortion. Across studies, the median (interquartile range) sample size of was 89 (22 to 209) participants. Of the included 18 studies, 12 (67%) had a prospective temporality. Fourteen studies (78%) were performed in endemic regions: Brazil (*n* = 7), Colombia (*n* = 3), French Polynesia (*n* = 1), French Guinea (*n* = 1), Martinique (*n* = 1) and Trinidad and Tobago (*n* = 1). The main characteristics of the included studies are displayed in Table 1.

**TABLE 1:** Main characteristics of the included studies.

Author and reference	N	Country and setting	Study design	Definition of suspected Zika virus infection	Imaging test	Reference standard	Conflict of interest	Exclusion criteria
Besnard (38)	19	French Polynesia Setting: Centre Hospitaller de Polynésie Franc̨aise (CHPF),private clinics and district hospitals	Retrospective	Symptoms of Zika during outbreak ZIKV RT PCR	US/Pre and Post	Postnatal anomalies	None declared	Toxoplasmosis, chromosomal abnormalities, hepatitis, rubella, syphilis
Rodó (24)	72	Spain **Setting:** Tertiary healthcare center	Prospective	Pregnant women that traveled to any of the endemic countries 8 weeks before her last menstrual period or during pregnancy; or pregnant women with sexual intercourse with partner that had traveled to any of the vectoral endemic countries in the past 6 months. Confirmed case: positive ZIKV RNA PCR in any maternal or fetal sample (serum, urine or amniotic fluid)	US/MRI	Newborns with clinical/radiological findings consistent of CZS Adverse perinatal outcome: abortion or miscarriage related to ZIKV or CZS.	None declared	Amniocentesis: • Toxoplasmosis, • CMV, • Parvovirus B19, • Herpes virus I and II, • Varicella Zoster. • Microarray was performed to rule out genetic conditions.
Schaub (1)	14	Martinique **Setting:** Tertiary healthcare center	Retrospective case series	Confirmed Zika virus fetal infection A positive ZIKV RT-PCR in at least one of the following samples: amniotic fluid, or fetal blood or from placenta, amnion, cerebrospinal fluid or brain after delivery.	US	Congenital brain abnormalities	None declared	• Chromosomal abnormalities • CGH array • Dengue and Chikungunya virus and CMV. • Toxoplasmosis and rubella infections were excluded by maternal serology.,
Sohan (40)	100	Trinidad y Tobago **Setting:** Public health center clinicians with interest in fetal medicine	Case series retrospective	Symptoms of Zika and Zika RT-PCR Anti Zika virus IgM antibodies in the absence of anti-dengue virus IgM and IgG antibodies	US	Examination at birth	None declared	RT PCR Dengue and Chikungunya
Mulkey (36)	82	Barranquilla, Colombia and (80) Washington United States (2) **Setting:** Clinical research facility in conjunction with Children’s National Health System	Prospective	Pregnant women with symptoms meeting Centers for Disease Control and Prevention clinical criteria for probable ZIKV infection, with or without confirmatory laboratory testing	US/MRI	Postnatal anomalies and examination	None declared	No
Sanz- Cortes (47)	214	Colombia **Setting.** Cediul- Cedifetal clinic in conjunction with Baylor College of Medicine	Prospective	Suspected Zika symptoms or their partners IgM Zika Zika RT-PCR	US/MRI	Postnatal anomalies and Examination	None declared	TORCH, chromosomal abnormalities History of smoking, mother BMI. Medication, chronic condition
Pomar (39)	301	French Guiana **Setting**: Three referral centers	Prospective	Pregnant women with positive IgM ZikZIKV RNA amplification and/or serology Confirmed congenital Zika virus infection either by Zika virus RNA amplification by RT-PCR by at least one fetal/neonatal sample Placenta, amniotic fluid, cerebrospinal fluid, urine or blood.	US	“Pregnancy outcome”	None declared	TORCH PCR, karyotype and CGH array. Hematologic and biochemical screening at birth
Sarno (32)	52	Salvador, Bahia Brazil **Setting:** Two public referral center and one private ultrasound training center	Prospective	Pregnant women with symptoms in endemic setting No Zika serology was carried out Fetal microcephaly related to ZIKV Infection with a molecular or epidemiological link to ZIKV: a. Confirmed case of Zikv b. Sexual contact with confirmed case c. History of symptoms or signs of ZIKV infection and was residing or traveling to endemic area while pregnant d. ZIKV present in amniotic fluid or present in fetal brain tissue	US	Postnatal anomalies	None declared	TORCH and chromosomal abnormalities No Zika serology was carried out
Pereira (34)	182	Rio de Janeiro, Brazil **Setting:** Tertiary maternal and pediatric referral center	Prospective	Women with a positive result for Zika virus confirmed by serum or urine reverse-transcription polymerase chain reaction (RT-PCR) who underwent at least one prenatal ultrasonography session after infection and had known neonatal outcomes	US	Composite adverse neonatal outcome defined as perinatal death (stillbirth or death within 28 days of life), an abnormal finding on neonatal examination or an abnormal finding on postnatal neuroimaging	Grant Reproductive Scientist	Serologic or molecular testing for dengue, chikungunya, Parvovirus, CMV, measles, syphilis, HIV. Exclusion criteria: chronic medical conditions
Melo (30)	11	Brazil Campina Grande, Paraiba **Setting.** Tertiary referral fetal medicine service	Prospective	Mothers with symptoms of ZIKV infection and with fetus with some brain abnormalities. Zika RT PCR in amniocentesis	US/MRI	Postnatal neuroimaging Clinical neonatal features Pathology findings	None declared	Diabetes and Chronic Hypertension were excluded in the mothers, and drug use, alcohol consumption, smoking, medication. TORCHS serology Test for other arbovirus were found to be negative by enzyme-linked immunosorbent assay and PCR examination in blood or amniotic fluid.
Nogueira (33)	216	São, Paulo Brazil **Setting.** Reference Hospital in Region	Prospective	Mothers with Zika symptoms identified by the city public health authority Blood sample collected during infection Zika RT PCR positive	US/MRI	Adverse birth outcomes	São Paulo Research Foundation	Toxo, HIV, rubella, cytomegalovirus, herpes simplex, syphilis (TORCHS)
Pires (31)	8	Brazil Pernambuco state	Retrospective of prospectively case series data	Mothers with presumed or confirmed Zika virus infection according to Brazilian Ministry Health guidelines	US/MRI	Brain abnormalities Birth outcome	None declared	TORCH and dengue virus serology, genetic abnormalities, primary microcephaly, and teratogens.
Carvalho (29)	22	Brazil Northeast region **Setting** Tertiary referral center	Retrospective	Diagnosis of fetal microcephaly or definite or highly probable diagnosis of maternal infection following classification recommended by the Brazilian Ministry of Health	US/MRI	Postnatal anomalies US and MRI and CAT	None declared	TORCH +, chromosomal abnormalities, syndromic microcephaly, teratogens Genetic clinical postnatal evaluation at birth to rule out genetic syndromes.
Walker et al. (39)	56	Columbia University Medical Center	retrospective	Pregnant women with recent ZIKV infection. Availability of ultrasound data within diagnosed with ZIKV infection after travel to countries with local transmission	US	femur sparring pattern of intrauterine growth restriction. conservative (AC < 3%, wZ-score e2) and traditional (AC < 10%, wZ-score e1.3) definition for IUGR to allow comparison of results with AC:FL, a fetal body ratio for AC normalized to FL.	None declared	No
Gutiérrez-Sánchez et al. (46)	209	Santader Colombia Tertiary referral center 3	prospective	Pregnant women were tested with RT- PCR to confirm or reject the diagnosis of ZIKV infection were included in the final analyses.	US	Brain abnormalities	Colciencias	Almost in 42% of patients they could not exclude other abnormalities.
Walker et al. (39)	95	Tertiary care center in Miami Florida United States	retrospective	Pregnant women were screened for possible ZIKV exposure. ZIKV RNA + in maternal, fetal, neonatal. Neonatal ZKV PCR urine, cerebrospinal fluid or serum	US	abnormal fetal growth trajectory using fetal indices (HC/FL ratio) neonates with ZIKV-associated anomalies and abnormal postnatal testing.	None	Preterm delivery 14%
Marbán-Castro et al. (37)	195	Spain Barcelona	Prospective and multicentric	Pregnant women that traveled endemic area or partner that travel. ZIKV RT PCR, IgG, IgM Zika antibodies Microneutralization titers > 1:32 indicative of antibodies ZikV	US	Adverse perinatal outcome Brain abnormalities Neurological assessment	“Centro de Excelencia Severo Ochoa 2019–2023” Program (CEX2018-000806-S), and support from the Generalitat de Catalunya through the CERCA Program.	Antibodies against Dengue and CHIKV
Coutinho (35)	511	Ribeirão Preto Brazil	Prospective population cohort study	Pregnant women were tested with RT- PCR in blood, urine, amniotic fluid or fetoplacental tissue to confirm diagnosis of ZIKV infection	US Prenatal/early neonatal data	Adverse perinatal outcome: Miscarriage and stillbirths Brain abnormalities with or without microcephaly Postnatal neuroimage studies and clinical follow up	Fundac̨ão de Apoio ao Ensino Pesquisa e Assistência do Hospital das Clínicas da Faculdade de Medicina de Ribeirão Preto e Centro de Vigilância Epidemiológica “Prof. Alexandre Vranjac”	Prenatal routine maternal testing was performed for toxoplasmosis, hepatitis B and C, human immunodeficiency virus, and syphilis. Maternal comorbidities, substance abuse and medications were recorded. Infants were subjected to testing for syphilis (treponemal and reaginic tests) and toxoplasmosis (IgM and IgG ELISA). Blood, saliva, urine, and/or liquor were tested for enterovirus, parvovirus, HHV-6, HSV-I, HSV-II, and CMV *via* PCR assays.

CAT, computerized axial tomography; CGH, comparative genomic hybridization; CMV, cytomegalovirus; CZS, congenital Zika syndrome; RT-PCR, reverse transcription polymerase chain reaction; MRI, magnetic resonance imaging; US, ultrasound.

The studies were published between 2016 and 2021, 6 were retrospective and 12 prospective studies. Among the eligible studies, there were a total of 2,359 fetuses; however, 36 (1.5%) women opted for termination of pregnancy.

Geographical distribution was as follows: studies in endemic settings: seven studies were performed in Brazil (29–35), three in Colombia (4, 36, 37), one in French Polynesia (38), one in French Guinea (39), one in Martinique (1), and one in Trinidad and Tobago (40). Two studies were performed in Barcelona, a non-endemic setting (41, 42). Two other studies were from the United States (43, 44). The variation in the number of pregnant women ranged from 8 (31) to 511 (35).

### Overview of diagnostic criteria used in the included studies

#### Brazil (endemic region)

Six out of the seven (86%) studies conducted in Brazil used definitions of presumed Zika infection, characterized by symptoms and/or serological Zika IgG/IgM in the absence of dengue and chikungunya antibodies. Confirmed Zika maternal infection was defined according to The Brazilian Ministry of Health and WHO guidelines. Two studies (32, 33) defined inclusion criteria as fetal microcephaly with a molecular or epidemiological link to ZIKV without other conditions known to cause microcephaly. One study included mothers with symptoms of ZIKV infection, whose fetuses had brain abnormalities (34).

#### Colombia (endemic region)

Two of the three (67%) studies conducted in Colombia were performed in the same city (Barranquilla). One of these two studies recruited participants from a clinical research facility with pregnant mothers with symptoms that met the guidelines from the CDC with or without confirmatory laboratory testing for ZIKV. The other study from Barranquilla included mothers with symptoms of infection or if their partners had symptoms, and anti-ZIKV IgM positive or ZIKV RT-PCR was required (4). The third study, conducted in Santander, used inclusion criteria similar to those used in above studies, enrolling women with symptoms of infection or if their partners had symptoms, and anti-ZIKV IgM positive or ZIKV RT-PCR was required.

#### Other endemic regions

Studies conducted in Trinidad and Tobago and French territories used similar inclusion criteria: pregnant women with positive serology for Zika virus antibodies, confirmation by RT-PCR ZIKV in at least one of the following biological materials: fetal/neonatal sample amniotic fluid, fetal blood, placenta, amnion, cerebrospinal fluid or brain tissue after delivery. Besides checking for antibodies of Dengue and Chikungunya, one study ruled out those infections with RT-PCR (35).

#### Europe (non-endemic region)

In two studies carried out in Spain.

#### Non-endemic region

In European studies, an inclusion criterion (41, 42) was a history of travel to an endemic area during pregnancy or 2 months before pregnancy. If the sexual partner had traveled to affected countries in the previous 6 months to the pregnancy, it was considered potential exposure to ZIKV. A positive RT-PCR test of ZIKV in serum and/or urine samples and/or a pregnant woman with a positive anti-ZIKV IgM antibodies, with a negative anti-dengue virus IgM antibodies and an anti-ZIKV neutralization titer ≥ 1/32 was a confirmation of infection.

Two retrospective cohorts were from the United States, one from New York and the other from Miami (43, 44). No informed consent was required in either. The definition of exposure to ZIKV infection was made following CDC guidelines. A confirmed cZIKV was reported with any positive nucleic acid test from a serum, urine, or cerebrospinal fluid sample. A positive or equivocal anti-ZIKV IgM antibody from an infant’s blood sample with a negative nucleic acid test was considered probable ZIKV infection.

#### Index test

Of the 18 included studies, 10 (56%) used prenatal ultrasound only, six studies (33%) evaluated fetuses with a combination of prenatal ultrasound and fetal MRI, and two studies (11%) added a postnatal MRI evaluation in addition to the prenatal ultrasound. Prenatal ultrasound included fetal biometry, detailed anatomy assessment and/or neuro-sonography performed by experienced sonographers, radiologists, or maternal fetal medicine specialists. Fetal MRI was performed in a total of six studies; two (4, 36) used 1.5T scanners and one (34) included a 3T MRI evaluation. Standard acquisition protocols were reported in the above studies, without use of maternal or fetal sedation. The MRI findings were interpreted by neuroradiologists and fetal radiologists. The remaining three studies did not report on the fetal MRI procedures or results.

In six studies the image test was performed between 16 and 25 weeks (1, 34–36, 38, 40) while four performed the test between 26 and 32.3 weeks (4, 31, 41, 42), and seven studies reported monthly scans (37, 38, 43–47). In the remaining study (39), the index test was performed between 18 and 33–34 weeks and stratified by trimester of gestation. More information on scans and definitions of anomalies is provided in the supplementary material (Tables SA1, SA2 Appendix 2 in Supplementary files).

#### Reference standard

A total of 10 studies reported that children underwent complete clinical examination and additional tests like cranial US, computed tomography, and ocular examination (4, 31, 33, 35, 37, 39–41, 43). Two studies reported just birth outcomes (gestational age at delivery, cesarean section delivery, and Apgar scores). One study reported a composite neonatal outcome, defined as perinatal death (stillbirth or death within 28 days of life), an abnormal finding on neonatal examination, or an abnormal finding in postnatal neuroimage (42). One study reported the outcome as a categorization of brain abnormalities (43). Four studies reported adverse perinatal outcomes, brain abnormalities and postnatal neurological assessment (38, 40, 45, 47). More information on definitions of reference standard is provided in Table SA2 Appendix 2 in Supplementary files).

#### Risk of bias

**FIGURE 2:**
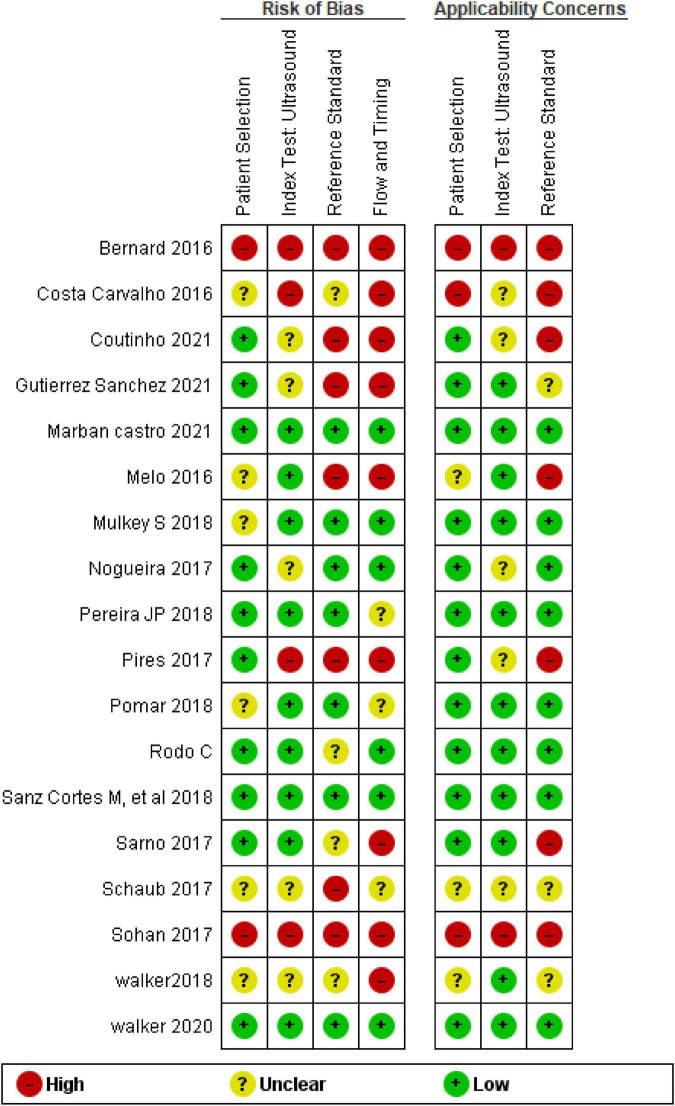
QUADAS 2 risk of bias and applicability of concerns.

Figure 2 summarizes the risk of bias assessment and applicability concerns in the 18 included studies. Only three (17%) of the 18 studies were considered at low risk of bias for all risk domains – including applicability concerns. Overall, a low risk of patient selection was found in 10 (56%) studies, a low risk of bias of index test in 9 (50%) studies, a low risk of bias of reference test in 7 (39%) and a low risk of bias in flow and timing in 6 (33%) studies.

#### Characteristics of the included studies in the meta-analysis

Sufficient outcome data were available for six studies involving 763 pregnant women. Five studies (83%) had a prospective temporality. The median sample was 82 participants (interquartile range: 64 to 226). Among the 763 participants, there were 168 (22%) cases identified with laboratory confirmed ZIKV.

Of the six studies with sufficient information for meta-analysis, one did not report the gestation age at which ultrasound imagining was captured. One study performed ultrasound monthly, and the four remaining studies performed ultrasound at a median (interquartile range) of 25.7 (22.6 to 27.4) weeks of gestational age (min = 20, max = 28.4). Fetal MRI was performed in four studies (6 with a total of 136 fetuses, in three studies, the median time for performing the fetal MRI was 28.1 (27.1 to 29.6) weeks of gestational age (Supplementary Table SA2).

**FIGURE 3:**
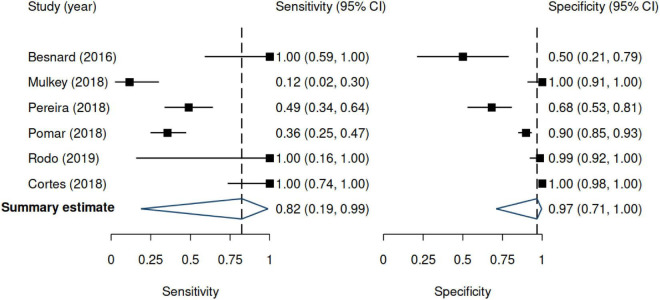
Forest plots for sensitivity and specificity. Results are based on the random-effects model. 95% CI denotes 95% confidence interval. The positive likelihood ratio was 24.9 (95% CI 2.11–293.2), and the negative likelihood ratio was 0.19 (95% CI 0.02–2.05).

#### Diagnostic accuracy of ultrasound

**FIGURE 4:**
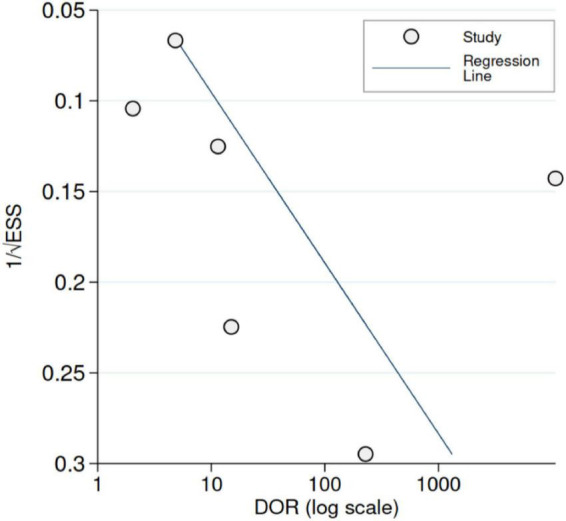
Funnel plot with Deek’s regression line [predicted diagnostic odds ratio (DOR)]. ESS denotes effective sample size. Deek’s test (*p* = 0.33).

Across studies, the sensitivity of US ranged from 12 to 100%, whereas the specificity ranged from 50 to 100%. Figure 3 shows the random-effects model summary estimates for sensitivity and specificity. There was evidence of statistical heterogeneity, indicating that the estimates varied more than expected by chance. The I^2^ was 87.6% for sensitivity and 92.1% for specificity (Cochran’s *Q*-test, *p* < 0.001 for both), indicating substantial heterogeneity. Deek’s test indicated no evidence of funnel plot asymmetry (*p* = 0.33) (Figure 4).

**FIGURE 5:**
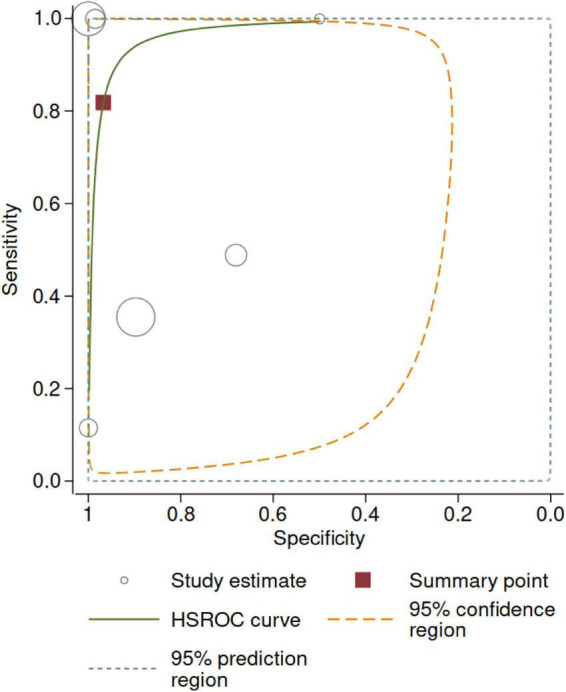
Hierarchical summary receiver operating curve (HSROC). The size of the circles is proportional to the number of participants in each study. The 95% confidence region represents the uncertainty around the summary point. The 95% prediction region represents the uncertainty regarding the estimate to be obtained in a new, well-conducted study—given the observed data (i.e., current levels of statistical heterogeneity).

Figure 5 shows the HSROC. The area under the HSROC was 0.97 (95% CI 0.72–1.00) and the summary DOR was 140 (95% CI 3.0–7564, *p* < 0.001). However, the predictive region showed large uncertainty regarding true accuracy to be estimated in a new study.

#### Certainty of the evidence (GRADE)

Table 2 summarizes the GRADE assessment based on the meta-analysis of 6 studies. Overall, the certainty of the evidence was “very low”, primarily because of the risk of bias, inconsistency, and imprecision.

**TABLE 2:** Summary of the main findings.

Test results	Number of results per 1,000 patients evaluated (95% CI)			Number of participants (studies)	Certainty of the evidence (GRADE)
	
	Prevalence 2% typically seen in	Prevalence 5% typically seen in	Prevalencia 15% typically seen in		
**True positive**	**16** (4 a 20)	**41** (10 a 50)	**123** (28 a 149)	168 (6)	⊕⁣○⁣○⁣○ **Very low**^a,b,c^
**False negative**	**4** (0 a 16)	**9** (0 a 40)	**27** (1 a 122)		
**True negative**	**951** (696 a 980)	**922** (675 a 950)	**825** (603 a 850)	595 (6)	⊕⁣○⁣○⁣○ **Very low**^1,d,e,f^
**False positive**	**29** (0 a 284)	**28** (0 a 275)	**25** (0 a 247)		

CI, confidence interval.

^a^Risk of bias: We downgraded the evidence by one level because of the overall high risk of bias. One study had a high risk of bias in all domains (38). Two studies had an unclear risk of bias in patient selection (36, 39). One study had unclear risk of bias for the reference standard (41). Two studies had unclear risk of bias for flow and timing (39, 34). Only one study had low risk of bias in all domains (4).

^b^Inconsistency: We downgraded the evidence by one level because of statistical heterogeneity.

^b^Imprecision: We downgraded the evidence by two levels, because the confidence interval was wide, and included clinically irrelevant diagnostic accuracy estimates.

^c^Publication bias: We did not downgrade the evidence for this criterion, since only 6 studies were included in the meta-analysis.

### Summary of evidence

Table 2 provides a summary of the main findings. Incorporated in the table are the explanations for grading of the evidence. We presented hypothetical testing outcomes for 1,000.

## Discussion

In this systematic review and meta-analysis, existing evidence on the diagnostic accuracy of prenatal image for congenital Zika syndrome was characterized by (1) substantial heterogeneity; (2) studies clustered in Brazil show that approximately half of the fetuses born out of ZIKV-infected mothers have abnormalities in the prenatal US imaging; (3) studies conducted around the world show no or minimal association between fetus prenatal imaging and the CZS hallmarks; (4) despite their heterogeneity, all studies confirmed the appearance of major anomalies of the central nervous system, including ventriculomegaly, cortical atrophy, calcifications, and anomalies of the corpus callosum; (5) less commonly reported abnormalities include intracranial calcifications at the gray matter–white matter junction, basal ganglia, and/or thalamus; cortical migrational abnormalities; skull with a collapsed appearance with overlapping sutures and redundant skin fold; intracranial herniation of orbital fat; and clot in the confluence of sinuses.

Studies clustered in Brazil show higher proportion of abnormalities related to CZS. The study by Sarno, Pires, Pereira, Carvalhlo, Brasil, and colleagues clearly states the proportion of CZS abnormalities ranging from a quarter up to 80% in some cases (32, 33, 41, 42). Of highlight is the report by Pomar et al. that one-third of cases show severe manifestation of CZS at birth or fetal loss. The remaining studies from Spain, France, Guyana, and Trinidad and Tobago describe lower proportion of abnormalities observed in prenatal imaging related to maternal ZIKV infection. These studies show fetus abnormalities in < 20% of cases.

### Strengths and limitations

This review has several strengths. The search strategies were sensitive, extensive, updated, and peer-reviewed by an information specialist. Then, the study protocol was prospectively designed and registered with PROSPERO in order to reduce bias.

This systematic review, like similar studies, has some limitations. During the meta-analysis, we compared pooled estimates between different study populations. None of the studies that were included provided head-to-head comparison between MRI and US.

### Interpretation of results

Caution in the interpretation of the results is warranted. In this study, there was a high proportion of studies at high risk of bias, and with high concern regarding applicability across all domains. These findings reduce the validity and applicability of the findings. The lack of standardization of definitions of brain abnormalities and lack of similar time follow-up are the limitations of the study. A plausible solution is conducting long-term follow-up studies to accurately assign newborns and infants suffering from CZS. The use of various instruments and algorithms to perform US or MRI definitively could affect the diagnosis of the cases.

### Future research

Long-term studies are currently warranted to clarify the clinical and developmental relevance of any CZS abnormality findings. Such studies should first clarify the role of imaging abnormalities in predicting CZS, as well as differentiating it from other congenital infections. This can be accomplished by elaborating diagnostic accuracy studies: performance of the index test in a standardized and blinded manner and ensuring that the reference standard is performed on all participants.

One plausible approach includes the use of artificial intelligence to accurately identify additional abnormalities that could suggest CZS. Also, long-term studies looking at postnatal neuroimaging in infants who had normal prenatal imaging could reveal new mild abnormalities. Perhaps, the term *congenital Zika syndrome* is preferable to refer to cases that include microcephaly and other clinical signs of this congenital malformation disorder. Such endeavor will help in defining combined findings from clinical, laboratory, imaging, and pathological examinations that provide a more complete picture of the severe damage and developmental abnormalities caused by ZIKV infection.

### Conclusion

The evidence supporting the accuracy of prenatal image for early diagnosis of congenital Zika syndrome remains low. The available evidence is in urgent need of development of standardized algorithms that could identify the whole spectrum of the disease. For now, we recommend the integration of prenatal and postnatal imaging and blood biomarkers to fully evaluate the risk assessment of fetus born to maternal infected women.

## Data availability statement

All coded data will be available upon reasonable request to corresponding author, provided the citation of this study. The protocol can be accessed at doi: 10.1097/MD9.0000000000000183.

## Author contributions

TH: conceptualization. TH, IC-B, AG, and TVP: data curation. TH, IC-B, and TVP: formal analysis. TH, IC-B, and AG: investigation and software. TH and TVP: methodology. TH: writing the original draft. IC-B, AG, and TVP: writing – review, and editing. All authors contributed to the article and approved the submitted version.
