# Supplementary material for: Diagnostic accuracy of prenatal imaging for the diagnosis of congenital Zika syndrome: Systematic review and meta-analysis
Source: Front Med (Lausanne). 2022 Sep 29;9:962765. doi: 10.3389/fmed.2022.962765 (PMC9556817; doi:10.3389/fmed.2022.962765)
Supplement: Supplementary file 1 [file Data_Sheet_1.pdf]

Diagnostic accuracy of prenatal imaging for congenital Zika syndrome: systematic review and meta-analysis

Appendix 1 Supplementary tables and figures

Table of contents

Table S1 SEDATE guidelines
Table S2 PRISMA DTA checklist
Table S3 QUADAS 2 tool risk of bias and applicability of concers signaling questions
Table S4 Excluded studies with reasons

CC BY SA

Table S1: Sedate guidelines

Section & Topic	No	Item	Reported on page #
**TITLE OR ABSTRACT**			
	**1**	Identification as a study of diagnostic accuracy using at least one measure of accuracy (such as sensitivity, specificity, predictive values, or AUC)	1
**ABSTRACT**			
	**2**	Structured summary of study design, methods, results, and conclusions (for specific guidance, see STARD for Abstracts)	2
**INTRODUCTION**			
	**3**	Scientific and clinical background, including the intended use and clinical role of the index test	4
	**4**	Study objectives and hypotheses	5
**METHODS**			
Study design	**5**	Whether data collection was planned before the index test and reference standard were performed (prospective study) or after (retrospective study)	5
Participants	**6**	Eligibility criteria	5
	**7**	On what basis potentially eligible participants were identified (such as symptoms, results from previous tests, inclusion in registry)	5
	**8**	Where and when potentially eligible participants were identified (setting, location and dates)	6
	**9**	Whether participants formed a consecutive, random or convenience series	6
Test methods	**10a**	Index test, in sufficient detail to allow replication	6
	**10b**	Reference standard, in sufficient detail to allow replication	6
	**11**	Rationale for choosing the reference standard (if alternatives exist)	6
	**12a**	Definition of and rationale for test positivity cut-offs or result categories of the index test, distinguishing pre-specified from exploratory	6
	**12b**	Definition of and rationale for test positivity cut-offs or result categories of the reference standard, distinguishing pre-specified from exploratory	6
	**13a**	Whether clinical information and reference standard results were available to the performers/readers of the index test	6
	**13b**	Whether clinical information and index test results were available to the assessors of the reference standard	6
Analysis	**14**	Methods for estimating or comparing measures of diagnostic accuracy	7
	**15**	How indeterminate index test or reference standard results were handled	9,10
	**16**	How missing data on the index test and reference standard were handled	9,10
	**17**	Any analyses of variability in diagnostic accuracy, distinguishing pre-specified from exploratory	8
	**18**	Intended sample size and how it was determined	
**RESULTS**			
Participants	**19**	Flow of participants, using a diagram	34
	**20**	Baseline demographic and clinical characteristics of participants	8
	**21a**	Distribution of severity of disease in those with the target condition	10,11
	**21b**	Distribution of alternative diagnoses in those without the target condition	11
	**22**	Time interval and any clinical interventions between index test and reference standard	11
Test results	**23**	Cross tabulation of the index test results (or their distribution) by the results of the reference standard	Supplementary material
	**24**	Estimates of diagnostic accuracy and their precision (such as 95% confidence intervals)	12,13
	**25**	Any adverse events from performing the index test or the reference standard	
**DISCUSSION**			

CC BY SA

	**26**	Study limitations, including sources of potential bias, statistical uncertainty, and generalisability	14
	**27**	Implications for practice, including the intended use and clinical role of the index test	14,15
**OTHER INFORMATION**			
	**28**	Registration number and name of registry	5
	**29**	Where the full study protocol can be accessed	16
	**30**	Sources of funding and other support; role of funders	16

STARD 2015

AIM

STARD stands for “Standards for Reporting Diagnostic accuracy studies”. This list of items was developed to contribute to the completeness and transparency of reporting of diagnostic accuracy studies. Authors can use the list to write informative study reports. Editors and peer-reviewers can use it to evaluate whether the information has been included in manuscripts submitted for publication.

EXPLANATION

A **diagnostic accuracy study** evaluates the ability of one or more medical tests to correctly classify study participants as having a **target condition.** This can be a disease, a disease stage, response or benefit from therapy, or an event or condition in the future. A medical test can be an imaging procedure, a laboratory test, elements from history and physical examination, a combination of these, or any other method for collecting information about the current health status of a patient.

The test whose accuracy is evaluated is called **index test.** A study can evaluate the accuracy of one or more index tests. Evaluating the ability of a medical test to correctly classify patients is typically done by comparing the distribution of the index test results with those of the **reference standard**. The reference standard is the best available method for establishing the presence or absence of the target condition. An accuracy study can rely on one or more reference standards.

If test results are categorized as either positive or negative, the cross tabulation of the index test results against those of the reference standard can be used to estimate the **sensitivity** of the index test (the proportion of participants *with* the target condition who have a positive index test), and its **specificity** (the proportion *without* the target condition who have a negative index test). From this cross tabulation (sometimes referred to as the contingency or “2x2” table), several other accuracy statistics can be estimated, such as the positive and negative **predictive values** of the test. Confidence intervals around estimates of accuracy can then be calculated to quantify the statistical **precision** of the measurements.

If the index test results can take more than two values, categorization of test results as positive or negative requires a **test positivity cut-off**. When multiple such cut-offs can be defined, authors can report a receiver operating characteristic (ROC) curve which graphically represents the combination of sensitivity and specificity for each possible test positivity cut-off. The **area under the ROC curve** informs in a single numerical value about the overall diagnostic accuracy of the index test.

The **intended use** of a medical test can be diagnosis, screening, staging, monitoring, surveillance, prediction or prognosis. The **clinical role** of a test explains its position relative to existing tests in the clinical pathway. A replacement test, for example, replaces an existing test. A triage test is used before an existing test; an add-on test is used after

CC BY SA

an existing test.

Besides diagnostic accuracy, several other outcomes and statistics may be relevant in the evaluation of medical tests. Medical tests can also be used to classify patients for purposes other than diagnosis, such as staging or prognosis. The STARD list was not explicitly developed for these other outcomes, statistics, and study types, although most STARD items would still apply.

DEVELOPMENT

This STARD list was released in 2015. The 30 items were identified by an international expert group of methodologists, researchers, and editors. The guiding principle in the development of STARD was to select items that, when reported, would help readers to judge the potential for bias in the study, to appraise the applicability of the study findings and the validity of conclusions and recommendations. The list represents an update of the first version, which was published in 2003.

More information can be found on http://www.equator-network.org/reporting-guidelines/stard.

Section/topic	#	PRISMA-DTA Checklist Item	Reported on page #
**TITLE / ABSTRACT**			
Title	1	Identify the report as a systematic review (+/- meta-analysis) of diagnostic test accuracy (DTA) studies.	1
Abstract	2	Abstract: See PRISMA-DTA for abstracts.	1
**INTRODUCTION**			
Rationale	3	Describe the rationale for the review in the context of what is already known.	2
Clinical role of index test	D1	State the scientific and clinical background, including the intended use and clinical role of the index test, and if applicable, the rationale for minimally acceptable test accuracy (or minimum difference in accuracy for comparative design).	1,2
Objectives	4	Provide an explicit statement of question(s) being addressed in terms of participants, index test(s), and target condition(s).	2
**METHODS**			
Protocol and registration	5	Indicate if a review protocol exists, if and where it can be accessed (e.g., Web address), and, if available, provide registration information including registration number.	1
Eligibility criteria	6	Specify study characteristics (participants, setting, index test(s), reference standard(s), target condition(s), and study design) and report characteristics (e.g., years considered, language, publication status) used as criteria for eligibility, giving rationale.	Table 1
Information sources	7	Describe all information sources (e.g., databases with dates of coverage, contact with study authors to identify additional studies) in the search and date last searched.	Figure 1
Search	8	Present full search strategies for all electronic databases and other sources searched, including any limits used, such that they could be repeated.	Supplementary material
Study selection	9	State the process for selecting studies (i.e., screening, eligibility, included in systematic review, and, if applicable, included in the meta-analysis).	8
Data collection process	10	Describe method of data extraction from reports (e.g., piloted forms, independently, in duplicate) and any processes for obtaining and confirming data from investigators.	6
Definitions for data extraction	11	Provide definitions used in data extraction and classifications of target condition(s), index test(s), reference standard(s) and other characteristics (e.g. study design, clinical setting).	5,6
Risk of bias and applicability	12	Describe methods used for assessing risk of bias in individual studies and concerns regarding the applicability to the review question.	7
Diagnostic accuracy measures	13	State the principal diagnostic accuracy measure(s) reported (e.g. sensitivity, specificity) and state the unit of assessment (e.g. per-patient, per-lesion).	12,13
Synthesis of results	14	Describe methods of handling data, combining results of studies and describing variability between studies. This could include, but is not limited to: a) handling of multiple definitions of target condition. b) handling of multiple thresholds of test positivity, c) handling multiple index test readers, d) handling of indeterminate test results, e) grouping and comparing tests, f) handling of different reference standards	14-16

Section/topic	#	PRISMA-DTA Checklist Item	Reported on page #
Meta-analysis	D2	Report the statistical methods used for meta-analyses, if performed.	12.13
Additional analyses	16	Describe methods of additional analyses (e.g., sensitivity or subgroup analyses, meta-regression), if done, indicating which were pre-specified.	
**RESULTS**			
Study selection	17	Provide numbers of studies screened, assessed for eligibility, included in the review (and included in meta-analysis, if applicable) with reasons for exclusions at each stage, ideally with a flow diagram.	8
Study characteristics	18	For each included study provide citations and present key characteristics including: a) participant characteristics (presentation, prior testing), b) clinical setting, c) study design, d) target condition definition, e) index test, f) reference standard, g) sample size, h) funding sources	Table 1
Risk of bias and applicability	19	Present evaluation of risk of bias and concerns regarding applicability for each study.	33
Results of individual studies	20	For each analysis in each study (e.g. unique combination of index test, reference standard, and positivity threshold) report 2x2 data (TP, FP, FN, TN) with estimates of diagnostic accuracy and confidence intervals, ideally with a forest or receiver operator characteristic (ROC) plot.	35-37
Synthesis of results	21	Describe test accuracy, including variability; if meta-analysis was done, include results and confidence intervals.	12,13
Additional analysis	23	Give results of additional analyses, if done (e.g., sensitivity or subgroup analyses, meta-regression; analysis of index test: failure rates, proportion of inconclusive results, adverse events).	
**DISCUSSION**			
Summary of evidence	24	Summarize the main findings including the strength of evidence.	32
Limitations	25	Discuss limitations from included studies (e.g. risk of bias and concerns regarding applicability) and from the review process (e.g. incomplete retrieval of identified research).	14
Conclusions	26	Provide a general interpretation of the results in the context of other evidence. Discuss implications for future research and clinical practice (e.g. the intended use and clinical role of the index test).	16
**FUNDING**			
Funding	27	For the systematic review, describe the sources of funding and other support and the role of the funders.	16

Adapted From: McInnes MDF, Moher D, Thombs BD, McGrath TA, Bossuyt PM, The PRISMA-DTA Group (2018). Preferred Reporting Items for a Systematic Review and Meta-analysis of Diagnostic Test Accuracy Studies: The PRISMA-DTA Statement. JAMA. 2018 Jan 23;319(4):388-396. doi: 10.1001/jama.2017.19163.

Table S3 QUADAS 2 tool Risk of bias and applicability of concer

Phase 3: Risk of bias and applicability judgments

QUADAS-2 is structured so that 4 key domains are each rated in terms of the risk of bias and the concern regarding applicability to the research question (as defined above). Each key domain has a set of signalling questions to help reach the judgments regarding bias and applicability.

DOMAIN 1: PATIENT SELECTION

A. Risk of Bias

Describe methods of patient selection:

- ❖ Was a consecutive or random sample of patients enrolled? Yes/No/Unclear
- ❖ Was a case-control design avoided? Yes/No/Unclear
- ❖ Did the study avoid inappropriate exclusions? Yes/No/Unclear

Could the selection of patients have introduced bias? RISK: LOW/HIGH/UNCLEAR

B. Concerns regarding applicability

Describe included patients (prior testing, presentation, intended use of index test and setting)**:**

Is there concern that the included patients do not match the review question? CONCERN: LOW/HIGH/UNCLEAR

CC BY SA

DOMAIN 2: INDEX TEST(S)

If more than one index test was used, please complete for each test.

A. Risk of Bias

Describe the index test and how it was conducted and interpreted:

- ❖ Were the index test results interpreted without knowledge of the results of the reference standard? Yes/No/Unclear
- ❖ If a threshold was used, was it pre-specified? Yes/No/Unclear

Could the conduct or interpretation of the index test have introduced bias? **RISK: LOW /HIGH/UNCLEAR**

B. Concerns regarding applicability

Is there concern that the index test, its conduct, or interpretation differ from the review question? **CONCERN: LOW /HIGH/UNCLEAR**

CC BY SA

DOMAIN 3: REFERENCE STANDARD

A. Risk of Bias

Describe the reference standard and how it was conducted and interpreted:

- ❖ Is the reference standard likely to correctly classify the target condition? — Yes/No/Unclear
- ❖ Were the reference standard results interpreted without knowledge of the results of the index test? — Yes/No/Unclear

Could the reference standard, its conduct, or its interpretation have introduced bias? — **RISK: LOW /HIGH/UNCLEAR**

B. Concerns regarding applicability

Is there concern that the target condition as defined by the reference standard does not match the review question? — **CONCERN: LOW /HIGH/UNCLEAR**

CC BY SA

DOMAIN 4: FLOW AND TIMING

A. Risk of Bias

Describe any patients who did not receive the index test(s) and/or reference standard or who were excluded from the 2x2 table (refer to flow diagram):

Describe the time interval and any interventions between index test(s) and reference standard:

- ❖ Was there an appropriate interval between index test(s) and reference standard? Yes/No/Unclear
- ❖ Did all patients receive a reference standard? Yes/No/Unclear
- ❖ Did patients receive the same reference standard? Yes/No/Unclear
- ❖ Were all patients included in the analysis? Yes/No/Unclear

Could the patient flow have introduced bias? **RISK: LOW /HIGH/UNCLEAR**

cc BY SA

Table S4. Excluded studies with reasons

PMID	**Reasons for Exclusions**	**Studies excluded after evaluation of the full paper**
**34287556**	Epidemiological study without prenatal image and part of the sample was published I another cohort	Soares de Oliveira 2016
**28332092**	Postmortem histopathology findings in congenital zika syndrome case series	Chimelli L,et.al 2017
**33395421**	All postnatal images studies for neonatal cohort	Souza 2021
**31968331**	Review of neurological abnormalities found in CT and MRI of fetus with zika congenital syndrome	Neimeyer 2020
**31949320**	There is no index test report.	Pereira AM 2020
**31896285**	Systematic review on postnatal neuroimaging- Anomalies evaluated Postnatally	Radaelli G 2020
**30365699**	There is no index test report. Anomalies evaluated postnatally.	Martins RS 2018
**29894797**	There is no index test report	Rodriguez -Morales 2018
**27509902**	There is no index test report	van der Linden 2016

CC BY SA

**29972474**	There is no index report : no prenatal image study was offered.	Fatima GV 2016
30817678	Anomalies Postnatally. CT brain scan and Histopathology	Aragao Fatima 2019
29412347	Excluded on Type of Study. Prevalence of Microcephaly	Ribeiro 2018
31340371	There is no index test report. Anomalies evaluated postnatally.	Roma 2018
30922930	Letter to the Editor	Santana 2019
2614330	Case report	Sarno 2016
**29030384**	No index test prenatally. Post natal Follow up CAT scans	Petribu 2017
**26943629**	Preliminary report of a cohort that had follow up study that we included- Included the last version of this cohort. (cohort from JP Pereira)	Brasil 2016
30424827	Target group: Congenital Brain abnormalities in patients with “exantema”	Kikuti 2018
**28594771**	Preliminary cohort reported by -duplicate population by Sanz Cortes	Parra Saavedra 2017
**27090801**	3 patients included in the report	Guillemmet 2016
**28329257**	Congenital infections a group them zika	Meneses
**28134669**	Review of neuroimaging findings and reported cases of congenital zika syndrome	Araujo Junior 2017
27960197.	No prenatal image information was retrieved from this article.	Honein 2017

**21853665**	Comparison between demographics and perinatal outcomes in patients that travel to endemic settings	Adhikari 2017
**29995729**	No prenatal image or index test was available	Conners 2018
29539287.	Patients enrolled in ZIKA-DFA-FE cohort study. And publish in another cohort	Hoen 2018
	Insufficient detail on outcomes	Rao
**34491004**	Correlation between cortical measurements and ophthalmological abnormalities in children	Daza 2021
**30985517**	Decide to exclude nature contact via telephone the patients “exposed to zika” and neonatal examinations	Calle-Giraldo 2019
**31954155**	Zika virus detection in amniotic fluid and Zika-associated birth defects.	Mercado M 2020
**31146294**	“Transient” population with Zika “PCR results” and “presumptive results”	Merriam 2020
**33872214**	Evaluation of a congenital zika program	Mulkey 2021
**34315277**	Population: children with postnatal neuroimage	Alves 2021
		Lima
**33684110**	Population: children with neonatal ultrasound assessment	Ximenes RAA 2021
	Protocol for congenital zika syndrome cohort	Buekens 2016
**3265434**	Population children with brain abnormalities no prenatal image test.	Sanders Pereira Pinto 2020
**27080092**	Newborns with radiological features	Cavalheiro 2016
**29661493**	Commentary to a paper	Yasri S 2018
**29242091**	No prenatal image or index test	De Araujo 2018
**27641777**	Seroprevalence study	De Araujo 2016
**27481629**	Review	Vouga 2016
**30760071**	No prenatal image data could be retrieved	Carpio-Orantes 2020
	Brain abnormalities in neonates without congenital zika syndrome	Mulkey 2020
**30939961**	Letter to the editor	Sookaromdee 2021
	Review article	Chen 2016

CC BY SA

	Case report	Moreira
**27028667**	Case report	Driggers 2016
**33068528**	Vertical transmission evaluated by a model	Ades 2021
**30914212**	Neurological abnormalities in children without prenatal image data	Melo 2020
**29197364**	Methodologic aspects for improve statistical power in assessing zika infection outcomes	Duarte EC 2017
**28976853**	Brain neonatal abnormalities	Mejdoubi M 2017
**27690200**	Review	Ticconi 2016
	Placenta infection French Guiana- same cohort published later and no prenatal image information	Pomar 2020
**27248295**	US National Registry data	Simeone 2016
**27352748**	Comment	Nicastri 2016
**30678125**	Infants with neuroimage results and clinical assesments	Lage 2019
**29284702**	Evaluation of congenital microcephaly in infants	Rick 2017
**26960750**	Letter to the editor	Villamil Gomez 2016
**28372868**	Letter to the editor	Garcia Robledo 2017
**29743756**	Editorial	Ribeiro BNF
**26959259**	Microcephaly epidemiology study	Texeira 2016
**31660908**	Children with microcephaly evaluated for eye damage.	de Paula Guimarães C 2019
**27906905**	Case reports MMwR report	Van der Linden 2016
	Case report	Moreira 2017
	French Guiana cohort published latera 2018	Pomar 2017

- de Araújo TVB, Rodrigues LC, de Alencar Ximenes RA, de Barros Miranda-Filho D, Montarroyos UR, de Melo APL, Valongueiro S, de Albuquerque MFPM, Souza WV, Braga C, Filho SPB, Cordeiro MT, Vazquez E, Di Cavalcanti Souza Cruz D, Henriques CMP, Bezerra LCA, da Silva Castanha PM, Dhalia R, Marques-Júnior ETA, Martelli CMT; investigators from the Microcephaly Epidemic Research Group; Brazilian Ministry of Health; Pan American Health Organization; Instituto de Medicina Integral Professor Fernando Figueira; State Health Department of Pernambuco. Association between Zika virus infection and microcephaly in Brazil, January to May, 2016: preliminary report of a case-control study. Lancet Infect Dis. 2018

- Honein MA, Dawson AL, Petersen EE, Jones AM, Lee EH, Yazdy MM, Ahmad N, Macdonald J, Evert N, Bingham A, Ellington SR, Shapiro-Mendoza CK, Oduyebo T, Fine AD, Brown CM, Sommer JN, Gupta J, Cavicchia P, Slavinski S, White JL, Owen SM, Petersen LR, Boyle C, Meaney-Delman D, Jamieson DJ; US Zika Pregnancy Registry Collaboration. Birth Defects Among Fetuses and Infants of US Women With Evidence of Possible Zika Virus Infection During Pregnancy. JAMA. 2017 Jan 3;317(1):59-68. doi: 10.1001/jama.2016.19006. PMID: 27960197.
- Rao R, Gaw SL, Han CS, Platt LD, Silverman NS. Zika Risk and Pregnancy in Clinical Practice: Ongoing Experience as the Outbreak Evolves. Obstet Gynecol. 2017 Jun;129(6):1098-1103. doi:
- Hoen B, Schaub B, Funk AL, Ardillon V, Boullard M, Cabié A, Callier C, Carles G, Cassadou S, Césaire R, Douine M, Herrmann-Storck C, Kadhel P, Laouénan C, Madec Y, Monthieux A, Nacher M, Najioullah F, Rousset D, Ryan C, Schepers K, Stegmann-Planchard S, Tressières B, Voluménie JL, Yassinguezo S, Janky E, Fontanet A. Pregnancy Outcomes after ZIKV Infection in French Territories in the Americas. N Engl J Med. 2018 Mar 15;378(11):985-994. doi: 10.1056/NEJMoa1709481.
- Calle-Giraldo JP, Rojas CA, Hurtado IC, Barco C, Libreros D, Sánchez PJ, López P, Arias A, Dávalos DM, Lesmes MC, Pinzón E, Ortiz VA, López-Medina E. Outcomes of Congenital Zika Virus Infection During an Outbreak in Valle del Cauca, Colombia. Pediatr Infect Dis J. 2019 Jul;38(7):735-740. doi: 10.1097/INF.0000000000002307.
- Merriam AA, Nhan-Chang CL, Huerta-Bogdan BI, Wapner R, Gyamfi-Bannerman C. A Single-Center Experience with a Pregnant Immigrant Population and Zika Virus Serologic Screening in New York City. Am J Perinatol. 2020 Jun;37(7):731-737. doi: 10.1055/s-0039-1688819. Epub 2019 May 30. PMID: 31146294.
- Mulkey SB, Ansusinha E, Cristante C, Russo SM, Biddle C, Kousa YA, Pesacreta L, Jantausch B, Hanisch B, Harik N, Hamdy RF, Hahn A, Chang T, Jaafar M, Ambrose T, Vezina G, Bulas DI, Wessel D, du Plessis AJ, DeBiasi RL. Complexities of Zika Diagnosis and Evaluation in a U.S. Congenital Zika Program. Am J Trop Med Hyg. 2021 Apr 19;104(6):2210-2219. doi: 10.4269/ajtmh.20-1256. PMID: 33872214; PMCID: PMC8176469.
- Ximenes RAA, Miranda-Filho DB, Montarroyos UR, Martelli CMT, Araújo TVB, Brickley E, Albuquerque MFPM, Souza WV, Ventura LO, Ventura CV, Gois AL, Leal MC, Oliveira DMDS, Eickmann SH, Carvalho MDCG, Silva PFSD, Rocha MAW, Ramos RCF, Brandão-Filho SP, Cordeiro MT, Bezerra LCA, Dimech G, Valongueiro S, Pires P, Castanha PMDS, Dhalia R, Marques-Júnior ETA, Rodrigues LC; Microcephaly Epidemic Research Group (MERG). Zika-related adverse outcomes in a cohort of pregnant women with rash in Pernambuco, Brazil. PLoS Negl Trop Dis. 2021 Mar 8;15(3)
- Garcia-Robledo JE, Rodriguez-Morales AJ. Fetal Imaging, Other Infectious Diseases Screening of Fetus With Zika Virus Infection and the Need for Long-Term Follow-Up. Pediatr Neurol. 2017 Jun;71:e1. doi: 10.1016/j.pediatrneurol.2017.02.012. Epub 2017 Mar 1. PMID: 28372868.
- Martins RS, Froes MH, Katz G, Sato APS. Epidemiological profile of a cohort of symptomatic pregnant women with suspected Zika virus infection in the State of São Paulo, Brazil, 2015-2018. Epidemiol Serv Saude. 2021 Jul 19;30(3):e2020827. English, Portuguese. doi: 10.1590/S1679-49742021000300011. PMID: 34287556.
- Chimelli L, Melo ASO, Avvad-Portari E, Wiley CA, Camacho AHS, Lopes VS, Machado HN, Andrade CV, Dock DCA, Moreira ME, Tovar-Moll F, Oliveira-Szejnfeld PS, Carvalho ACG, Ugarte ON, Batista AGM, Amorim MMR, Melo FO, Ferreira TA, Marinho JRL, Azevedo GS, Leal JIBF, da Costa RFM, Rehen S, Arruda MB, Brindeiro RM, Delvechio R, Aguiar RS, Tanuri A. The spectrum of neuropathological changes

associated with congenital Zika virus infection. Acta Neuropathol. 2017 Jun;133(6):983-999. doi: 10.1007/s00401-017-1699-5. Epub 2017 Mar 22. PMID: 28332092.

- Souza JP, Méio MDBB, de Andrade LM, Figueiredo MR, Gomes Junior SC, Pereira Junior JP, Brickley E, Lopes Moreira ME. Adverse fetal and neonatal outcomes in pregnancies with confirmed Zika Virus infection in Rio de Janeiro, Brazil: A cohort study. PLoS Negl Trop Dis. 2021 Jan 4;15(1):e0008893. doi: 10.1371/journal.pntd.0008893. PMID: 33395421; PMCID: PMC7781387.
- Niemeyer B, Hollanda R, Muniz B, Marchiori E. What We Can Find Beyond the Classic Neuroimaging Findings of Congenital Zika Virus Syndrome? [published online ahead of print, 2020 Jan 22]. *Eur Neurol*. 2020;1–8.
- Pereira AM, Araujo Júnior E, Werner H, Monteiro DLM. Zika Virus and Pregnancy: Association between Acute Infection and Microcephaly in Newborns in the State of Rio de Janeiro, Brazil. *Geburtshilfe Frauenheilkd*. 2020;80(1):60–65.
- Radaelli G, Lahorgue Nunes M, Bernardi Soder R, et al. Review of neuroimaging findings in congenital Zika virus syndrome and its relation to the time of infection.
- Rodriguez-Morales AJ, Cardona-Ospina JA, Ramirez-Jaramillo V, et al. Diagnosis and outcomes of pregnant women with Zika virus infection in two municipalities of Risaralda, Colombia: Second report of the ZIKERNCOL study. *Travel Med Infect Dis*. 2018;25:20–25.
- Martins RS, Fróes MH, Saad LDC, et al. Descriptive report of cases of congenital syndrome associated with Zika virus infection in the state of São Paulo, Brazil, from 2015 to 2017. *Epidemiol Serv Saude*. 2018;27(3):e2017382. Published 2018 Oct 22. doi:10.5123/S1679-49742018000300012
- van der Linden V, Filho EL, Lins OG, et al. Congenital Zika syndrome with arthrogryposis: retrospective case series study. *BMJ*. 2016;354:i3899. Published 2016 Aug 9. doi:10.1136/bmj.i3899
- França GVA, Pedi VD, Garcia MHO, Carmo GMID, Leal MB, Garcia LP. Congenital syndrome associated with Zika virus infection among live births in Brazil: a description of the distribution of reported and confirmed cases in 2015-2016 [published correction appears in Epidemiol Serv Saude. 2018 Aug 20;27(2):e20172062].
- de Fatima Viana Vasco Aragão M, van der Linden V, Petribu NC, Valenca MM, Parizel PM, de Mello RJV. Congenital Zika Syndrome: The Main Cause of Death and Correspondence Between Brain CT and Postmortem Histological Section Findings From the Same Individuals. *Top Magn Reson Imaging*. 2019;28(1):29–33. doi:10.1097/RMR.0000000000000194
- Ribeiro IG, Andrade MR, Silva JM, et al. Microcephaly in Piauí, Brazil: descriptive study during the Zika virus epidemic, 2015-2016. Microcefalia no Piauí, Brasil: estudo descritivo durante a epidemia do vírus Zika, 2015-2016. *Epidemiol Serv Saude*. 2018;27(1):e20163692. Published 2018 Feb 1. doi:10.5123/S1679-49742018000100002
- Roma JHF, Alves RC, Silva VSD, Ferreira MJ, Araújo C, Pavoni JHC. Descriptive study of suspected congenital Zika syndrome cases during the 2015-2016 epidemic in Brazil. *Rev Soc Bras Med Trop*. 2019;52:e20190105. Published 2019 Jul 18. doi:10.1590/0037-8682-010
- Santana MB, Lamas CC, Athayde JG, Calvet G, Moreira J, De Lorenzo A. Congenital Zika syndrome: is the heart part of its spectrum?. *Clin Microbiol Infect*. 2019;25(8):1043–1044. doi:10.1016/j.cmi.2019.03.0205-2019
- Sarno M, Sacramento GA, Khouri R, et al. Zika Virus Infection and Stillbirths: A Case of Hydrops Fetalis, Hydranencephaly and Fetal Demise. *PLoS Negl Trop Dis*. 2016;10(2):e0004517. Published 2016 Feb 25. doi:10.1371/journal.pntd.0004517
- Brasil P, Pereira JP Jr, Moreira ME, et al. Zika Virus Infection in Pregnant Women in Rio de Janeiro. *N Engl J Med*. 2016;375(24):2321–2334. doi:10.1056/NEJMoa1602412

- Kikuti Mariana, Cardoso Cristiane W., Prates Ana P.B., Paploski Igor A.D., Kitron Uriel, Reis Mitermayer G., Mochida Ganeshwaran H., Ribeiro Guilherme S.. Congenital brain abnormalities during a Zika virus epidemic in Salvador, Brazil, April 2015 to July 2016. Euro Surveill. 2018;23(45):pii=1700757. https://doi. org/10.2807/1560-7917.ES.2018.23.45.1700757
- Guillemette-Artur P, Besnard M, Eyrolle-Guignot D, Jouannic JM, Garel C. Prenatal brain MRI of fetuses with Zika virus infection. *Pediatr Radiol*. 2016;46(7):1032-1039.
- Meneses JDA, Ishigami AC, de Mello LM, et al.. Lessons Learned at the Epicenter of Brazil's Congenital Zika Epidemic: Evidence From 87 Confirmed Cases. Clin Infect Dis 2017;65(8):1431-33
- Soares de Oliveira-Szejnfeld P, Levine D, Melo AS, Amorim MM, Batista AG, Chimelli L, Tanuri A, Aguiar RS, Malinger G, Ximenes R, Robertson R, Szejnfeld J, Tovar-Moll F. Congenital Brain Abnormalities and Zika Virus: What the Radiologist Can Expect to See Prenatally and Postnatally. Radiology. 2016 Oct;281(1):203-18. doi: 10.1148/radiol.2016161584. Epub 2016 Aug 23. PMID: 27552432.
- Araujo Júnior E, Carvalho FH, Tonni G, Werner H. Prenatal imaging findings in fetal Zika virus infection. Curr Opin Obstet Gynecol. 2017 Apr;29(2):95-105. doi: 10.1097/GCO.0000000000000345. PMID: 28134669.
- Mercado M, Ailes EC, Daza M, Tong VT, Osorio J, Valencia D, Rico A, Galang RR, González M, Ricaldi JN, Anderson KN, Kamal N, Thomas JD, Villanueva J, Burkel VK, Meaney-Delman D, Gilboa SM, Honein MA, Jamieson DJ, Ospina ML. Zika virus detection in amniotic fluid and Zika-associated birth defects. Am J Obstet Gynecol. 2020 Jun;222(6):610.e1-610.e13. doi: 10.1016/j.ajog.2020.01.009. Epub 2020 Jan 15. PMID: 31954155; PMCID: PMC7477618.
- Sookaromdee P, Wiwanitkit V. Zika virus infection in pregnant women in Mexico. J Matern Fetal Neonatal Med. 2021 Jan;34(2):310
- C Lage ML, Carvalho AL, Ventura PA, Taguchi TB, Fernandes AS, Pinho SF, Santos-Junior OT, Ramos CL, Nascimento-Carvalho CM. Clinical, Neuroimaging, and Neurophysiological Findings in Children with Microcephaly Related to Congenital Zika Virus Infection. Int J Environ Res Public Health. 2019 Jan 23;16(3):309. doi: 10.3390/ijerph16030309. PMID: 30678125; PMCID: PMC6388186.
- Nicastri E, Castilletti C, Di Caro A, Capobianchi MR, Ippolito G. Diagnosis of Zika virus infection in pregnant women travelling to or residing in endemic areas. Lancet Infect Dis. 2016 Jul;16(7):771-772. doi: 10.1016/S1473-3099(16)30074-3. PMID: 27352748.
- Rick AM, Domek G, Cunningham M, Olson D, Lamb MM, Jimenez-Zambrano A, Heinrichs G, Berman S, Asturias EJ. High Background Congenital Microcephaly in Rural Guatemala: Implications for Neonatal Congenital Zika Virus Infection Screening. Glob Health Sci Pract. 2017 Dec 28;5(4):686-696. doi: 10.9745/GHSP-D-17-00116. PMID: 29284702; PMCID: PMC5752614.
- Villamil-Gómez WE, Mendoza-Guete A, Villalobos E, González-Arismendy E, Uribe-García AM, Castellanos JE, Rodríguez-Morales AJ. Diagnosis, management and follow-up of pregnant women with Zika virus infection: A preliminary report of the ZIKERNCOL cohort study on Sincelejo, Colombia. Travel Med Infect Dis. 2016 Mar-Apr;14(2):155-8. doi: 10.1016/j.tmaid.2016.02.004. Epub 2016 Mar 4. PMID: 26960750.
- Ribeiro BNF. Congenital Zika syndrome and neuroimaging findings. Radiol Bras. 2018 Mar-Apr;51(2):VII-VIII. doi: 10.1590/0100-3984.2018.51.2e2. PMID: 29743756; PMCID: PMC5935421.
- de Paula Guimarães C, Macedo MS, Barbosa MA, Marques SM, Costa PS, de Oliveira ÊC. Clinical findings in congenital infection by Zika virus: a retrospective study in a reference hospital in Central-West Brazil. BMC Pediatr. 2019 Oct 29;19(1):389. doi: 10.1186/s12887-019-1762-6. PMID: 31660908; PMCID: PMC6819383.
- Teixeira MG, Costa Mda C, de Oliveira WK, Nunes ML, Rodrigues LC. The Epidemic of Zika Virus-Related Microcephaly in Brazil: Detection, Control, Etiology, and Future Scenarios. Am J Public Health. 2016 Apr;106(4):601-5. doi: 10.2105/AJPH.2016.303113. PMID: 26959259; PMCID: PMC4816003.

- van der Linden V, Pessoa A, Dobyns W, Barkovich AJ, Júnior HV, Filho EL, Ribeiro EM, Leal MC, Coimbra PP, Aragão MF, Verçosa I, Ventura C, Ramos RC, Cruz DD, Cordeiro MT, Mota VM, Dott M, Hillard C, Moore CA. Description of 13 Infants Born During October 2015-January 2016 With Congenital Zika Virus Infection Without Microcephaly at Birth - Brazil. MMWR Morb Mortal Wkly Rep. 2016 Dec 2;65(47):1343-1348. doi: 10.15585/mmwr.mm6547e2. PMID: 27906905.
-
