# Supplementary material for: Diagnostic accuracy of prenatal imaging for the diagnosis of congenital Zika syndrome: Systematic review and meta-analysis
Source: Front Med (Lausanne). 2022 Sep 29;9:962765. doi: 10.3389/fmed.2022.962765 (PMC9556817; doi:10.3389/fmed.2022.962765)
Supplement: Supplementary file 2 [file Data_Sheet_2.pdf]

Diagnostic accuracy of prenatal imaging for diagnosis of congenital Zika syndrome: systematic review and meta-analysis

Appendix 2: Supplementary tables

- **Table of Contents**

- Search Strategy
- Table SA1. Characteristics of the Index Test in 18 included studies
- Table SA2. Population, Index Test, Reference Standard, Outcome in studies included
- Table SA3. Table 2 x 2 for studies in the meta analysis

- Definition of Brain anomalies
- Table SA4 Brain anomalies in patients with congenital zika syndrome

Search zika march 1 2022

(

SearchActionsDetailsQueryResultsTime#20
Search: **"zika virus infection"[MeSH Terms] OR "zika virus infection"[Title/Abstract] OR ("Congenital zika virus infection"[Title/Abstract] OR "zika infection"[Title/Abstract] OR "zika fever"[Title/Abstract] OR "zika virus disease"[Title/Abstract]) AND (" ultrasound"[Title/Abstract] OR "magnetic resonance"[Title/Abstract] OR "diagnostic image"[Title/Abstract]) OR "obstetric, ultrasonography"** Sort by: **Most Recent**
27713:28:25#19
Search: **((("zika infection"[Title/Abstract]) OR ("zika fever"[Title/Abstract])) OR (zika virus infection[Title/Abstract])) AND ((("ultrasonography"[Title/Abstract]) OR ("obstetric ultrasound"[Title/Abstract])) OR ("diagnostic imaging"[Title/Abstract]))** Sort by: **Most Recent**
2113:25:46#18
Search: **((((((("zika virus infection"[MeSH Terms]) OR (zika virus infection[Title/Abstract])) OR ("Congenital zika syndrome"[Title/Abstract])) OR ("Congenital zika virus infection"[Title/Abstract] AND (y_10[Filter]))) OR ("Congenital zika virus infection"[Title/Abstract])) OR ("zika infection"[Title/Abstract])) OR ("zika fever"[Title/Abstract])) OR ("zika virus disease"[Title/Abstract])) AND (((prenatal ultrasound[Title/Abstract]) OR ("magnetic resonance"[Title/Abstract])) OR ("diagnostic image"[Title/Abstract]))** Sort by: **Most Recent**
6913:23:38#17

Search: **((zika virus infection[Title/Abstract]) OR ("Congenital zika virus infection"[Title/Abstract])) AND ((("ultrasonography"[Title/Abstract]) OR ("obstetric ultrasound"[Title/Abstract])) OR ("diagnostic imaging"[Title/Abstract]))** Sort by: **Most Recent**
2113:21:54#16
Search: **(("zika infection"[Title/Abstract]) OR ("zika fever"[Title/Abstract])) AND (((prenatal ultrasound[Title/Abstract]) OR ("magnetic resonance"[Title/Abstract])) OR ("diagnostic image"[Title/Abstract]))** Sort by: **Most Recent**
513:05:27#15
Search: **(("Congenital zika syndrome"[Title/Abstract]) OR ("zika virus disease"[Title/Abstract])) AND ((("ultrasonography"[Title/Abstract]) OR ("obstetric ultrasound"[Title/Abstract])) OR ("diagnostic imaging"[Title/Abstract]))** Sort by: **Most Recent**
1513:03:45#14
Search: **(((("zika virus infection"[MeSH Terms]) AND (zika virus infection[Title/Abstract])) OR ("Congenital zika virus infection"[Title/Abstract])) OR ("zika virus disease"[Title/Abstract])) OR (((prenatal ultrasound[Title/Abstract]) OR ("magnetic resonance"[Title/Abstract])) OR ("diagnostic image"[Title/Abstract]))** Sort by: **Most Recent** 403,03912:44:00#13
Search: **(("Congenital zika virus infection"[Title/Abstract]) OR ("zika virus infection"[MeSH Terms])) AND (((prenatal ultrasound[Title/Abstract]) OR ("magnetic resonance"[Title/Abstract])) OR ("diagnostic image"[Title/Abstract]))** Sort by: **Most Recent**
5912:38:48#12
Search: **(((((("zika virus infection"[MeSH Terms]) OR (zika virus infection[Title/Abstract])) OR ("Congenital zika syndrome"[Title/Abstract])) OR ("Congenital zika virus infection"[Title/Abstract] AND (y_10[Filter])))) AND ((("ultrasonography"[Title/Abstract]) OR ("obstetric ultrasound"[Title/Abstract])) OR ("diagnostic imaging"[Title/Abstract]))) OR ((("ultrasonography"[Title/Abstract]) OR ("obstetric ultrasound"[Title/Abstract])) OR ("diagnostic imaging"[Title/Abstract]))) OR (((prenatal ultrasound[Title/Abstract]) OR ("magnetic resonance"[Title/Abstract])) OR ("diagnostic image"[Title/Abstract]))** Sort by: **Most Recent**
507,72512:37:32#11
Search: **((prenatal ultrasound[Title/Abstract]) OR ("magnetic resonance"[Title/Abstract])) OR ("diagnostic image"[Title/Abstract])** Sort by: **Most Recent**
401,48612:20:36#10
Search: **(("ultrasonography"[Title/Abstract]) OR ("obstetric ultrasound"[Title/Abstract])) OR ("diagnostic imaging"[Title/Abstract])** Sort by: **Most Recent**
119,92412:19:04#9

Search: **((((((("zika virus infection"[MeSH Terms]) OR (zika virus infection[Title/Abstract])) OR ("Congenital zika syndrome"[Title/Abstract])) OR ("Congenital zika virus infection"[Title/Abstract] AND (y_10[Filter]))) OR ("Congenital zika virus infection"[Title/Abstract])) OR ("zika infection"[Title/Abstract])) OR ("zika fever"[Title/Abstract])) OR ("zika virus disease"[Title/Abstract])** Sort by: **Most Recent**
6,54511:48:14#8
Search: **"zika virus disease"[Title/Abstract]** Sort by: **Most Recent**
24511:46:18#7
Search: **"zika fever"[Title/Abstract]** Sort by: **Most Recent**
11211:43:41#6
Search: **"zika infection"[Title/Abstract]** Sort by: **Most Recent**
28911:42:30#4
Search: **"Congenital zika virus infection"[Title/Abstract]** Sort by: **Most Recent**
13711:41:28#5
Search: **"Congenital zika virus infection"[Title/Abstract]** Filters: **in the last 10 years** Sort by: **Most Recent**
13711:41:21#3
Search: **"Congenital zika syndrome"[Title/Abstract]** Sort by: **Most Recent**
53611:38:43#2
Search: **zika virus infection[Title/Abstract]** Sort by: **Most Recent**
1,71611:37:28#1
Search: **"zika virus infection"[MeSH Terms]** Sort by: **Most Recent**
6,00011:36:39

Google

Congenital zika syndrome 506

"congenital zika infection" OR " ZikV congenital" AND "prenatal ultrasound" OR "imaging" OR "diagnostic "

Scopus 125

INDEXTERMS("zika virus infection") OR TITLE-ABS("zika virus infection") OR (TITLE-ABS("Congenital zika virus infection") OR TITLE-ABS("zika infection") OR TITLE-ABS("zika fever") OR TITLE-ABS("zika virus disease")) AND (TITLE-ABS(" ultrasound") OR TITLE-ABS("magnetic resonance") OR TITLE-ABS("diagnostic image")) OR "obstetric, ultrasonography". 125

Table SA1.Characteristics of the Index Test in the eighteen included studies

Author	Sample size	Index Test	ga US(w)	US assesments	Sample size MRI	ga MRI	Operators US: definition of the index test US	Blinded operators	Equipment MRI	Interpretation MRI
Besnard2016	19	US/MRI	NR	Not clear	7	29.1	Expert ob gyn, nurse	Not cleared	NR	NR
Sarno2017	52	US	24.1	20-24w<br>26-30w<br>After 34w	NA	NA	Fetal medicine	Blinded	NA	NA
Rodo2019	72	US/MRI	20.0	monthly	37	Third trimester	Detailed anatomy scan and neurosonograms	Not reported	NR	NR
Schaub2017	14	US	16-24	16-20w (12)<br>20-24w (10)<br>24-28w<br>(5)<br>>28w (4)	NA	NA	ISUOG guidelines, certified operators	Not cleared	NA	NA
Sohan2017	100	US	20	Not clear	NA	NA	ISUOG guidelines Clinician interest in fetal medicine	Blinded	NA	NA
Mulkey2018	82	US/MRI	25.1	Second and third trimester	82	25.1 and 30w	Radiologist after on-site protocol standardization	Blinded	1.5T<br>Single shot, fast spin, echo T2 weighed images	Neuroradiologist and Fetal radiologist
SanzCortes2018	214	US/MRI	28.4 w	monthly	10	30.1 +- 4.6w	Obstetricians and maternal fetal medicine specialists	Blinded	1.5T<br>No maternal or fetal sedation	Baylor College of Medicine
Pomar2018	301	US	Random	monthly	NA	NA	Radiology Unit with experienced sonographers. International and national guidelines	NA	NA	NA
Pereira2018	182	US	26.3w	Not clear	NA	NA	Perinatologist certified by FEBRASGO. Detailed anatomy scan	Blinded	NA	NA
Melo2018	11	US/MRI	22.0w	Not clear	6	NR	Fetal Medicine	NR	3T MRI or	NR

				about gestational age			specialists		1.5T with 8 channel body coil and standard acquisition protocols	
Nogueira2018	216	US/MRI	NR	Not clear	25	NR	Multidisciplinary medical team	NR	NR	NR
Pires2017	8	US/MRI	31.3w	Not clear	Postnatal	postnatal MRI	Board certified operator<br>ISUOG Guidelines	NR	NR	NR
Carvahlo2016	22	US/MRI	32.3w	Not clear	19	Postnatal MRI	Prenatal Guidelines ISUOG	NR	NR	NR
Walker2018	56	US	a. 18-23 6/7 w (19)<br>b. 24-27 6/7 w (17)<br><br>c. 28-33 6/7w (38)<br>d. >34w (46)	monthly	NA	NA	Guidelines IG 21 standards	NR	NA	NA
Gutierrez Sanchez2021	209	US	NR	Not clear	NA	NA	Certified Obstetrician and Maternal Fetal Medicine specialist. ISUOG guidelines	NR	NA	NA
Walker2020	95	US	27.0w +- 7.6w	monthly	7	Postnatal MRI if Postnatal cranial US show anomalies	Intergrowth 21 standards	Blinded analysis	NR	NR
MarbanCAstro 2021	195	US	NR	monthly	NA	NA	Experienced examiners prenatal and neursosonograms	NR	NA	NA
Coutinho2021	511	US	NR	monthly	NA	NA	NR	NR	NA	NA

Table SA2.Characteristics of 18 included studies CZS: PICO format											
Setting				Population					Index text		Reference standard
Study	year	study period	study temporality	inclusion criteria	Suspected Maternal ZK	Definition of Maternal Zika Infection	GA at inclusion(weeks)	GA at birth(weeks)	Definition of CZS	Number CZS fetuses	Included outcomes
Besnard	2016	2014-2015	Retrospective cohort	Pregnancies during zika outbreak with fetus with brain abnormalities	19	Zika symptoms and ZIKV RT PCR in amniotic fluid ,cerebral anomalies	34	31.4	Prenatal brain abnormalities in US and MRI in Zika confirmed fetus	7	Perinatal death, Neonatal unit admission, postnatal image anomalies and clinical evidence of brainstem dysfunction
Rodo	2019	2016-2017	Prospective cohort	Pregnant women epidemiological link	72	Confirmed case: serum and urine ZIKV RT PCR+ "Probable case" ZIKV IgM +/ZIKV IgG+ or indetermined PRNT	20	33.5	Prenatal brain abnormalities in US and MRI. Zika confirmed Serum or urine Zika PCR	2	Newborns with clinical/radiological findings consisted with congenital zika syndrome Adverse perinatal outcome: abortion or miscarriage related to ZIKV or CZS.
Mulkey	2018		Prospective cohort	Pregnant women with symptoms	82	Zika laboratory confirmation	25.1	38.4	Prenatal brain abnormalities US and MRI No laboratory zika confirmation was possible	3	Newborns with clinical/ radiological findings consistent of CZS
Sanz Cortes	2018	2015-2016	Prospective	Pregnant women with symptoms or if their partners had symptoms. Women diagnose with congenital anomalies compatible with CZS	214	Maternal symptoms And IgM Zika Zika RT-PCR	21.9	37.8	Prenatal brain abnormalities US and MRI Confirmed Zika	12	Perinatal outcomes: neonatal anthropometry, neurologic status Postnatal brain images (transcranial US, CT scanning and MRI) and Laboratory confirmation: RT PCR +maternal, amniotic fluid, placental or umbilical cord
Pomar	2018	2016-2017	Prospective	Pregnant patients with Zika positive serology or positive PCR Zika	301	Confirmed congenital ZIKV infection: +Zika RT-PCR at least in one sample (placenta, amniotic fluid, urine, blood) or Zika virus igM in umbilical cord/neonatal blood or in cerebrospinal fluid. Maternal contamination was excluded with IgM status on day 3 Umbilical cord samples from fetal loss	monthly	Not clear	Prenatal brain abnormalities in US	76	Clinical neonatologist, anthropometric measurements, neurological status. Four categories of CZS. A. Asymptomatic B. Mild or moderate C. Severe c D. Fetal loss and stillbirth E. "Any adverse outcome"
Gutierrez Sanchez	2021	2015-2017	Prospective	Pregnant patients with clinical symptoms with Zika PCR	209	Confirmed congenital zika infection: + Zika RT-PCR in blood, urine or amniocentesis sample Negative case: Zika RTPCR negative	31.5	38	Prenatal brain anomalies in US and Neurosonography and Neonatal Zika confirmed cases (Zika RT PCR)	32	Intrauterine Growth restriction, Birth outcome, apgar score.
Walker	2020	2016-2017	Retrospective	Pregnant women screening Questionnaire. Tertiary care center in Miami	95	Confirmed maternal infection: +Zika RT-PCR in blood; ZIKV IgM + or equivocal; ZIKV PRNT >10 titer Negative ZIKV IgM, positive or equivocal dengue IgM and ZIKV PRNT >10 titer	29.8	27.4	US Confirmed case:Positive ZIKV RT PCR in neonatal serum, urine or cerebrospinal fluid.	24	Clinical neonatal evaluation, postnatal US, MRI if indicated and additional auditory screening and visual testing. Fetal body ratio(HC:FL) at 10 centile and at 3erd centile
Walker	2018	2016-2017	Retrospective	Pregnant women from an area with local ZIKV transmission at Columbia Medical Center screening	56	Confirmed maternal infection: +Zika RT-PCR in blood; ZIKV IgM + or equivocal; ZIKV PRNT >10 titer Negative ZIKV IgM, positive or equivocal dengue IgM and ZIKV PRNT >10 titer	NR	37.4	US Confirmed case:Positive ZIKV RT PCR in neonatal serum, urine or cerebrospinal fluid Probable case: IgM Zika positive or equivocal with negative ZIKV RT PCR	3	Clinical neonatal evaluation, postnatal US, MRI if indicated and additional auditory screening and visual testing. Brain anomalies, Intrauterine growth restriction Fetal body ratio (HC:FL;AC:FL) <10percentile
Pereira	2018	2015-2016	Prospective	Pregnant women with ZIKV RT-PCR Blood /urine that underwent 1 prenatal US	182	Confirmed case: serum and urine ZIKV RT PCR+	26.3	38.6	Abnormal ultrasound: at least one of these parameters: any structural anomaly, abnormal fetal growth measurements (fetal growth restriction or macrosomia), abnormal umbilical artery, abnormal MCA doppler measurements, abnormal amniotic fluid assessment or pllacentomegaly. Categorization Zika related anomaly and another with unknown significance with Zika	37	Composite perinatal outcome defined by perinatal death , an abnormal finding of neonatal examination or abnormal finding on postnatal neuroimage Perinatal outcomes: NICU admission, Clinical examinations were performed by neonatologist, pediatric infectious disease specialist, geneticists, and neurologists

Table Characteristics of included studies CZS metanalysis											
Setting				Population					Index text		Reference standard
Study	year	study period	study temporality	inclusion criteria	Suspected Maternal ZK	Definition of Maternal Zika Infection	GA at inclusion(weeks)	GA at birth(weeks)	Definition of CZS	Number CZS fetuses	Included outcomes
Nogueira	2018	2016	Prospective cohort	Pregnant women with acute Zika symptoms	216	ZIKV RT-PCR positive	NR	37.5	US Prenatal brain abnormalities<br>ZIKV RT-PCR positive umbilical cord blood and urine.	18	Newborns with clinical/ radiological findings consistent of CZS<br>Adverse outcomes: brain anomalies, premature birth and abnormal otoacustic emission
Pires	2017	2015-2016	Retrospective	Pregnant women with confirmed or presumed Zika infection	8	Maternal symptoms<br>And<br>IgM Zika or<br>Zika RT-PCR according to Brazilian Ministry of Health	31.3	38.0	US<br>Prenatal brain abnormalities<br>Confirmed ZIKV PCR +	8	Birth outcomes: neonatal<br>Postnatal brain images (transcranial US, CT scanning and MRI) and Laboratory confirmation:<br>RT PCR + amniotic fluid, placental or umbilical cord
Schaub	2017	2016	Retrospective	Pregnant women with Zika Infection in their first or second trimester and with fetal brain anomalies	42	Confirmed ZKV RT-PCR maternal blood or positive Zika Virus IgM (ratio >3) or IgG seroconversion confirmed by Zika virus neutralization	16	27.4	Confirmed congenital zika infection: Zika virus RNA RT-PCR at fetal/neonatal sample, placenta, amniotic fluid, cerebrospinal fluid, urine and blood	14	Brain anomalies and autopsy
Sohan	2017	2016	Retrospective	Pregnant women with serologic evidence of Zika Infection (RT-PCR + and IgM + (2) and with symptoms	100	Confirmed maternal infection: +Zika RT-PCR in blood; ZIKV IgM + or equivocal; ZIKV PRNT >10 titer Negative ZIKV IgM, positive or equivocal dengue IgM and ZIKV PRNT >10 titer	20	27.6	No laboratory zika confirmation on fetal/neonatal sample were performed	6	Birth outcomes, Brrain anomalies
Melo	2016	2015-2016	Prospective	Pregnant women with Zika symptoms in the first half of pregnancy and fetus with some brain anomalies	11	Confirmed or presumed zika infection according to Brazilian Ministry of Health guidelines	22	39.0	Suspected cases: Brain anomalies, maternal history of Zika symptoms and strong IgG anti zika<br>Confirmed:<br>ZIKV RT-PCR + in amniotic fluid or neonatal sample	11	Amniotic fluid, Cord Blood, placenta, neonatal tissues collected postmortem
Carvahlo	2016	2015-2016	Retrospective	Pregnant women with suspected l zika infection and with probable fetus with microcephaly	22	Defiinite cases: laboratory evidence of zika virus infection (igM or ZIKV RT-PCR +) Guidelines Brazilian Ministry of Health	32.3	NR	Confirmed:<br>ZIKV RT-PCR + in amniotic fluid or neonatal sample	19	Birth outcome, Brain postnatal anomalies assessment with CT scans and MRI<br>Perinatal death
Marban Castro	2021	2016-2020	Prospective	Pregnant women that travelled to endemic areas or partner	195	Confirmed maternal infection: +Zika RT-PCR in blood; ZIKV IgG IgM Zika antibodies Microneutralization titers >1:32	NR	38.5	Confirmed case:Positive ZIKV RT PCR in neonatal serum, urine or cerebrospinal fluid<br>Probable case: IgM Zika positive or equivocal with negative ZIKV RT PCR	4	Clinical neonatal evaluation, postnatal US, MRI if indicated and additional auditory screening and visual testing.<br>Brain anomalies
Sarno	2017	2015-2016	Prospective	Pregnant women with zika symptoms	60	Molecular or epidemiological link with ZIKV: RT-PCR +, sexual contact with confirmed case, history of symptoms; residing or travelling to an area with ongoing transmission	24.1	NR	Confirmed cade:ZIKV RT-PCR + amniocentesis or fetal brain tissue	52	Clinical neonatal evaluation and postnatal medically indicated cranial ultrasound,eye examination)
Coutinho	2018	2015-2016	Prospective	Pregnant women with ZIKV RT-PCR Blood /urine	511	Pregnant women with ZIKV RT-PCR Blood /urine	NR	39	Anti IgM ZIKV antibodies +; ZIKA RNA +<br>Brain anomalies: neonatal neurologi dysfunction<br>Newborns with clinical/radiological findings consisted with congenital zika syndrome	19	Adverse perinatal outcome: abortion or miscarriage related to ZIKV or CZS<br>Stillbirth<br>Severe Brain anomalies<br>Neonatal deaths

Tables 2 x 2 – Studies included in meta-analysis

Besnard 2016

Population assessed in the study was comprised by 19 cases of congenital cerebral malformations. CZS was not examined for every case “For some cases with of non-microcephalic, congenital abnormalities we were not able to prove or exclude ZIKV infection retrospectively”. Data extracted took into account malformation and report of ZIKV infection, based on PCR evaluation or mother reporting symptoms during pregnancy.

	**N**	**Source**
TP (abnormal prenatal imaging + zika confirmation)	7	According to figure 4, cases with abnormal prenatal US and confirmation of ZIKV infection (4) and abnormal prenatal US+ mother experiencing symptoms of ZIKV infection + medical termination of pregnancy (3).
FP (abnormal prenatal imaging + zika negative)	6	According to figure 4, cases with abnormal prenatal US and ZIKV RT-PCR negative (3) and abnormal prenatal US+no symptom (3; cases 7, 9 and 12).
FN (normal prenatal imaging + abnormal postnatal outcome)	0	There is no report of normal prenatal US and brain abnormalities related to ZIKV infection.
TN (normal prenatal and mothers reporting no ZIKV infection symptoms/no ZIKVneutralising antibodies)	6	According to figure 4, prenatal US did not show brain abnormalities in 6 cases, 5 cases reported under group 3 and 1 under group 1b. It is mentioned that cases on group 3 had no ZIKV neutralising antibodies and no symptoms during pregnancy and laboratory tests were normal for the 1 case on group 1b, and the mother was asymtomatic.

Carvalho 2016

	N	Source
TP (abnormal prenatal imaging + zika confirmation or exclusion of other congenital infections)	19	The study reported 19 cases of microcephaly, ALL detected by prenatal US. Thus, FN and TN = zero.<br>According to the study:<br>ï "a direct association between fetal malformations and Zika virus could not be established as placenta investigations were not performed",<br>ï "In our series other congenital infections such as TORCH and syphilis were ruled out in all pregnant women, and genetic syndromes were excluded by clinical examination by a medical geneticist at birth."<br>ï "A limitation of our series is that only cases with CNS malformations detected on prenatal ultrasound were included. Thus, the congenital anomalies detected in our series may possibly represent the end-point of the spectrum of intrauterine Zika infection."<br>Thus, all cases were classified as TP.
FP (abnormal prenatal imaging + zika negative)	0	
FN (normal prenatal imaging + abnormal postnatal outcome)	0	
TN (normal prenatal and normal postnatal outcome)	0	

Mulkey 2018

	N	Source
TP (abnormal prenatal imaging + postnatal confirmation)	3	Study included 82 pregnant women with Zika virus infection, 3 cases of severe fetal brain abnormalities were prenatal imaging (TP). From the 79 cases with normal prenatal imaging, postnatal imaging was perfomed only in 61 cases to confirm CSZ. Abnormal postnatal imaging was observed in 23 newborn (FN). TN: 61 - 23 = 38
FP (abnormal prenatal imaging + no postnatal confirmation)	0	
FN (normal prenatal imaging + abnormal postnatal imaging)	23	
TN (normal prenatal and postnatal imaging)	38	

Nogueira 2017

	N	Source
TP (abnormal prenatal US + ZIKV RNA detected)	18	Data was extracted from Supplementary Table 5 (abnormal prenatal US) and Supplementary Table 6 (confirmation of CSZ - Abnormal neurological evaluation). Abnormal prenatal US was observed in the whole population (ZIKV-positive and ZIKV-negative).
FP (abnormal prenatal US + No ZIKV RNA detected)	0	
FN (normal prenatal US + ZIKV RNA detected)	0	
TN (normal prenatal US + No ZIKV RNA detected)	36	

Pereira 2018

	N	Source
TP (abnormal ultrasonography + abnormal neonatal outcome)	22	Data extracted from:<br>Figure. Flow of Patients Through the Study
FP (abnormal ultrasonography + normal neonatal outcome)	15	
FN (normal ultrasonography + abnormal neonatal outcome)	23	
TN (normal ultrasonography + normal neonatal outcome)	32	

Pomar 2018

	N	Source
TP (abnormal prenatal US + Confirmed congenital Zika virus infection)	27	cZIKV positive babies that had abnormal prenatal US listed on appendix 4 (15 cZIKV positive babies had normal prenatal US that were substracted from the 42 cZIKV positive babies – figure 2)
FP (abnormal prenatal US + Negative fetal/neonatal Zika virus testing)	22	cZIKV negative babies with abnormal prenatal US (appendix 4)
FN (normal prenatal US + Confirmed congenital Zika virus infection)	49	15 cZIKV positive babies that had normal prenatal US (appendix 4) + 33 cZIKV positive babies that were asymptomatic (figure 2)
TN (normal prenatal US + Negative fetal/neonatal Zika virus testing)	193	5 normal prenatal US (appendix 4) + 188 Negative fetal/neonatal Zika virus testing that were asymptomatic

Sanz Cortez 2018

	N	Source
TP (abnormal prenatal US + CZS positive cases)	12	All 12 fetuses showed significant brain findings during MRI and ultrasound evaluation.
FP (abnormal prenatal US + negative CZS)	0	CSZ was not confirmed for 202 subjects that were initially referred. The study did not mention any information on US findings for this group.
FN (normal prenatal US + CZS positive cases)	0	All 12 fetuses showed significant brain findings during MRI and ultrasound evaluation, no observation of normal US in CZS positive cases.
TN (CZS negative with normal US)	202	CSZ was not confirmed for 202 subjects that were initially referred. The study did not mention any information on US findings for this group.

Sarno 2017

	N	Source
TP (abnormal prenatal imaging + fetus classified as having ZIKV-related microcephaly after birth)	35	The study reports abnomal ultrasound findings of 52 fetuses with Zika virus-related microcephaly. Follow-up data were available in 35 of the 52 fetuses classified as having ZIKV-related microcephaly according to WHO criteria (TP).
FP (abnormal prenatal imaging + fetus not classified as having ZIKV-related microcephaly after birth)	17	
FN (normal prenatal imaging + fetus classified as having ZIKV-related microcephaly after birth)	0	
TN (normal prenatal imaging + fetus not classified as having ZIKV-related microcephaly after birt)	0	

Rodo 2019

	N	Source
TP (abnormal prenatal US + CZS positive cases)	2	In the "confirmed infection" group there were two cases of congenital disease (page 633.e6) and "MRI confirmed the abnormal ultrasound findings in the two CZS cases (p 0.042) (please see supplementary material)."
FP (abnormal prenatal US + no CZS)	1	Second-trimester ultrasound was normal in all except three cases: two cases of brain malformations in the confirmed group (2/8, 25%), both consistent with CZS (case 1 and 8, Table 2), **<u>and one case of congenital heart defect in the probable infection group</u>** (p = 0.004).
FN (normal prenatal US + CZS positive cases)	0	Only 2 CZS cases that were confirmed by US.
TN (CZS negative with normal US)	68	CZS was not confirmed in 7 out of 9 on "confirmed infection" and in 62 on the "Probable infection" group (1 case had abnormal US)

Schaub 2017

	N	Source
TP (abnormal prenatal imaging + zika confirmation)	14	The study reported 14 cases of pregnant women with confirmed Zika virus infection AND fetal abnormalities of the brain. Abnormal ultrasound scans was reported for ALL cases. Positive Zika virus RT-PCR is reported for ALL fetuses.
FP (abnormal prenatal imaging + zika negative)	0	
FN (normal prenatal imaging + zika confirmation)	0	
TN (normal prenatal and no zika confirmation)	0	

Definitions of fetal brain anomalies

Definitions of the anomalies in fetal brain reported: Three studies classify fetal brain findings in major and minor anomalies (37, 43, 46). Major anomalies associated to CZS included: major CNS abnormality, like agenesia of corpus callosum, brainstem dysgenesis and migration disorders. Minor findings included: abnormal doppler, umbilical artery and middle cerebral artery; fetal growth restriction (FGR), amniotic fluid anomalies, oligohydramnios, and polyhydramnios; choroid plexus cyst. One of the studies did not report any brain abnormalities definitions at all (35).

Fetal microcephaly: Mild to moderate microcephaly was determined based on the head circumference, but the criterion varied across studies. Of the 18 included studies, 6 (33.3%) used a head circumference smaller than three standard deviations below the mean to define microcephaly. One study defined severe microcephaly as a head circumference smaller than three standard deviations below the mean of the gestational age or smaller than two standard deviations below the mean, along with some other CNS findings. Five studies used the CDC definition of microcephaly as a head circumference smaller than the 3rd percentile for gestational age (33). Different HC growth curves were used to classify neonatal microcephaly: INTERGROWTH-21st (IG-21) (4, 47, 49), Fenton curves (36), IG-21 and the World Health Organization (WHO) Fetal Growth chart (WHO-FGC) (29,48). Sanz Cortes, et al compare neonatal head circumferences with both IG-21 and Fenton curves (4).

Fetal ventriculomegaly: Fetal ventriculomegaly definition varied across the studies. Majority of the studies classified ventriculomegaly as severe, above 15mm (4, 36-40). Other studies classified as a mild ventriculomegaly when the level of the atria measures between 10-12mm; Sarno *et al* (33), classified a mild anomaly when atria <15mm.One study used to cut off >10mm to describe ventriculomegaly (42).

Growth abnormalities: Pires *et al* estimated fetal weight (EFW) with the Hadlock 3 formula (39); Pereira, Mulkey defined FGR as fetal weight <10 percentile with Hadlock formula (35,42) ; IG-21 was used by (4,36) . One study reported femur sparring pattern of fetal growth restriction using fetal body ratios and IG-21 and did not use EFW to make the definition (47). Pereira small for gestational age neonate (SGA) was defined by IG-21 standards-Doppler studies. The pulsatility index of the umbilical artery and middle cerebral artery (MCA) were defined as abnormal if 1) greater than the 95th percentile or 2) if the peak systolic velocity of the MCA was greater than 1.5 multiples of the median

Table SA4. Brain anomalies in confirmed congenital zika syndrome

Study	Confirmed CZS	Ventriculomegaly severe	Dysgenesia callosal	Periventricular Calcifications	Migration disorders	Microcephaly	Cortical anomalies	Decreased brain volume	Heterotopia Nodular	Others
Besnard	19	7 (37%)	7(37%)	8 (42%)	6 (31%)	6 (31%)				
Mulkey	3	1				2				
Sanz Cortes	12		12 (100%)	11(92%)		7/9 (78%)	8/9 (89%)	√	√	
Pomar	76			5		3				
Gutierrez Sánchez	32		16	8	9	25				
Walker 2018	3					3				
Walker 2020	24					6				
Pereira	37	6 (16%)	2 (5%)	9 (24%)		7 (19%)				
Nogueira	18	NR	NR	NR	NR	NR	NR	NA	NA	
Pires	8			√√	√	8 (100%)				
Schaub	14	8	10 (71%)	10 (71%)		9				
Sohan	9	2					2			3 (enlarged cisterna magna)
Melo	11	10 (90%)	1			9 (82%)				
Carvahlo	19	10 (53%)		15 (79%)		17 (89%)				
Marban Castro	4									1 (subependymal pseudocyst)
Sarno	52	48(65%)	2	23 (44%)	NR	46 (88%)	NR	NA	NA	
Coutihno	20	√		√		7 (35%)	√	√		
Rodo	2					2				
